# The Skytrain plate and tectonic evolution of southwest Gondwana since Jurassic times

**DOI:** 10.1038/s41598-020-77070-6

**Published:** 2020-11-17

**Authors:** Graeme Eagles, Hannes Eisermann

**Affiliations:** Alfred Wegener Institut, Helmholtz Zentrum Für Polar Und Meeresforschung, Am Alten Hafen 26, 27568 Bremerhaven, Germany

**Keywords:** Geology, Geophysics, Tectonics

## Abstract

Uncertainty about the structure of the Falkland Plateau Basin has long hindered understanding of tectonic evolution in southwest Gondwana. New aeromagnetic data from the basin reveal Jurassic-onset seafloor spreading by motion of a single newly-recognized plate, Skytrain, which also governed continental extension in the Weddell Sea Embayment and possibly further afield in Antarctica. The Skytrain plate resolves a nearly century-old controversy by requiring a South American setting for the Falkland Islands in Gondwana. The Skytrain plate’s later motion provides a unifying context for post-Cambrian wide-angle paleomagnetic rotation, Cretaceous uplift, and post-Permian oblique collision in the Ellsworth Mountains of Antarctica. Further north, the Skytrain plate’s margins built a continuous conjugate ocean to the Weddell Sea in the Falkland Plateau Basin and central Scotia Sea. This ocean rules out venerable correlation-based interpretations for a Pacific margin location and subsequent long-distance translation of the South Georgia microcontinent as the Drake Passage gateway opened.

## Introduction

A region’s geological and tectonic evolution is most readily and reliably interpretable within the context of a self-consistent plate kinematic model. The lack of consensus on such a context for the modern regions that started as southwest Gondwana in Jurassic times stems from, and has sustained, uncertainties and controversies concerning the interpretations of regional geological correlations and large paleomagnetically-determined rock rotations from scattered outcrops at the margins of today’s South Atlantic Ocean, Weddell, and Scotia seas^1–5^. The associated uncertainties hinder attempts to better understand wider issues including the mechanisms of supercontinent breakup^6,7^, and the roles of regional Toarcian magmatism and basin anoxia^8^, or Paleogene oceanic gateway development in Drake Passage^4,9^, in global environmental and biotic change on geological timescales.

### Mobile continental blocks

**Figure 1:**
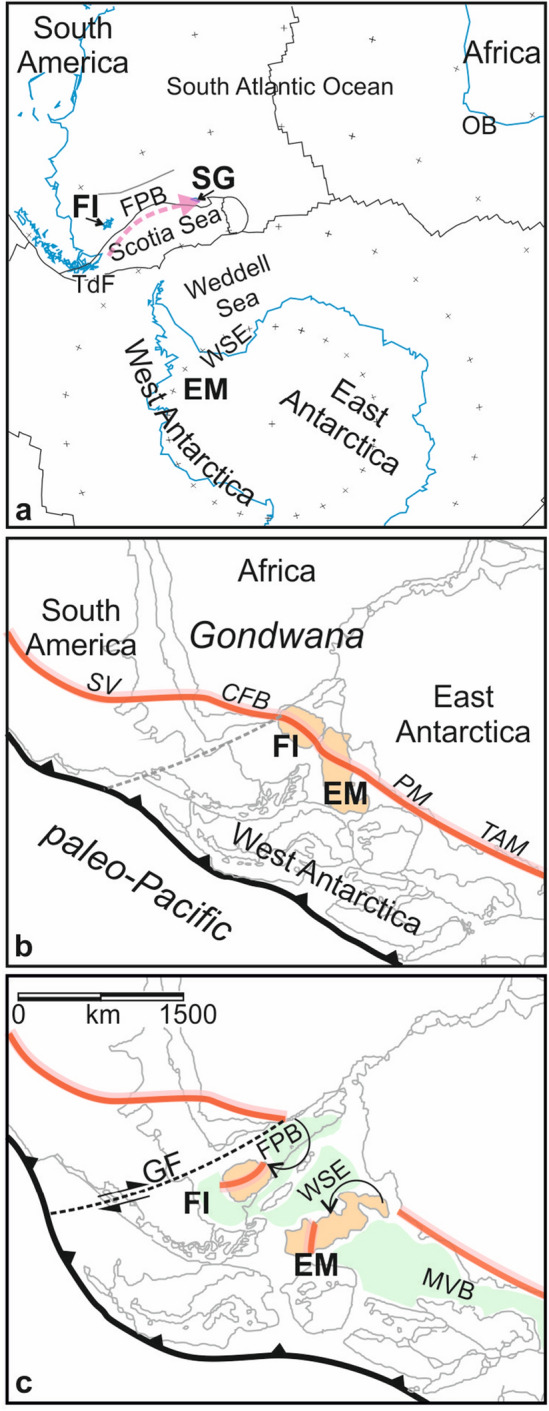
(**a**) Present-day plate tectonic context of the study region. Map and coastline data: Generic Mapping Tools^20^; active plate boundaries from a recent compilation^21^. Pink arrow: proposed geological correlation-based translation path of South Georgia (SG), along the southern edge of the Falkland Plateau Basin (FPB). *EM* Ellsworth Mountains, *FI* Falkland Islands; *OB* Outeniqua Basin; *TdF* Tierra del Fuego; *WSE* Weddell Sea Embayment. (**b**,**c**) Sketches of a geological correlation-based model^7^ for the initial stages of continental breakup at the southwest margin of Gondwana between Triassic (top) and mid-Jurassic (bottom) times. Red line: Gondwanide Orogen comprising the Cape Fold Belt (CFB), Pensacola Mountains (PM) Sierra de la Ventana (SV) and Transantarctic Mountains (TAM). Black arrows represent large opposing-sense rotations of the Falkland Islands and Ellsworth Mountains. *GF* Gastre Fault; *MVB* Mesozoic Victoria Basin.

Three of the most widely discussed sets of regional correlations are those concerning the continental blocks of South Georgia, the Falkland Islands and the Ellsworth Mountains (Fig. 1).

By the mid-1960s, long-known correlations of the Jurassic–mid Cretaceous rocks of the islands of South Georgia to those of Tierra del Fuego, which today are distant neighbours at opposite ends of the Scotia Sea (Fig. 1a), came to be framed as records of the growth, filling, and subsequent tectonic closure of a late Jurassic back-arc basin^2,10^. Since then, the correlations have been considered strong enough to constrain the basin and the islands of South Georgia to a Jurassic location immediately east of Tierra del Fuego. This requires the islands to have later translated eastwards by 1600 km to their present location. Much of this translation has been attributed to the action of strike-slip faults embedded within the arc plate above an Oligo-Miocene aged subduction zone fringing the Scotia Sea, whose rollback governed an intricate pattern of synchronously-active back-arc basins opening northwest-southeast in the west Scotia Sea, east–west in the East Scotia Sea, and north–south in the central Scotia Sea^2,9,11,12^.

Motions of continental blocks bearing the Ellsworth Mountains and Falkland Islands (Fig. 1) were first suggested in the mid-twentieth century on the basis of pioneering long-distance geological correlation studies that built on comparative observations dating back to the infancy of the continental drift debate^1^. Both were correlated sedimentologically and structurally to the Cape Fold Belt of South Africa and its neighbours in South America and Antarctica, the Sierra de la Ventana and the Pensacola and Transantarctic Mountains^13,14^. The correlations suggested the Permian–Triassic construction of a ~ 8000 km-long linear deformation belt, the Gondwanide Orogen, built by plate convergence at the subduction dominated paleo-Pacific margin of Gondwana (Fig. 1b). To restore the orogen so that its structures show a consistently continentward direction of tectonic transport requires the Falkland Islands to have rotated clockwise by 120° and drifted by 1000 km westwards prior to the opening of the South Atlantic^13^, and the Ellsworth Mountains to have moved 800 km southwestwards and have rotated anticlockwise by 90° since Cambrian times^14^ (Fig. 1c). Paleomagnetic work on the Falkland Islands and Ellsworth Mountains has delivered evidence for large rock rotations consistent with these proposed motions^15–19^.

Despite their long history, southwest Gondwana’s large block motions have all been questioned, and alternative but less kinematically-prescriptive regional correlation schemes have been suggested.

South Georgia’s late Jurassic and early Cretaceous rocks and structures do not preserve strong signals of Oligo-Miocene volcanism and faulting in the subduction zone that is supposed to have accommodated its eastwards translation. Instead, the islands’ tectonostratigraphy has been recast in terms of an intra-Gondwanan Jurassic-onset continental extensional margin^22^. Similarly, marine geophysical data from the neighbouring central Scotia Sea do not strongly or unequivocally support the occurrence of north–south directed back-arc spreading that is supposed to have accompanied rollback of the Oligo-Miocene trench. Instead, the central Scotia Sea has been presented as a fragment of the Jurassic and early Cretaceous ocean that accreted to the South Georgian margin^23^.

Both before and ever since their South African associations were emphasized, the Paleozoic rocks of the Falkland Islands have been alternatively correlated to those in the fill sequences of South American intracontinental basins^24,25^. The islands’ paleomagnetic rotations are only recorded reliably in a single-aged population of northeast-striking dykes. Whilst interpretable in terms of a large plate rotation having occurred very rapidly^26^, this has also been related to tectonic deformation of the dykes synchronous with their intrusion^27^ close to their present-day location in relation to South America. In contrast, the Ellsworth Mountains’ paleomagnetic history is more robustly interpretable in terms of plate motion because it has been repeatedly observed in multiple widely-distributed Cambrian lithologies and locations^18,19^. Despite this, the mountains’ precise location in Gondwana remains a matter of controversy related to the absence of evidence for a pre-Gondwanide Paleozoic phase of deformation known from the Cape Fold Belt and Pensacola Mountains^18,28^.

### The Falkland Plateau Basin

The Falkland Plateau is a submarine plateau that stretches 1200 km east of the Falkland Islands into the South Atlantic. Its southern edge has been proposed as the site of an Oligo-Miocene subduction zone along which South Georgia moved eastwards from Tierra del Fuego. Further north within the plateau interior lies a 500 km-wide sedimentary basin, the Falkland Plateau Basin, which is proposed to have opened synchronously with the Weddell Sea Embayment to accommodate the rotations of both the Falklands and Ellsworth blocks (Fig. 1c)^6,7,29–31^. Jurassic-aged reconstructions built to show the basin opening in response to these rotations require plate divergence within it to have been transformed westwards into southern Patagonia, causing several hundred kilometres of strike-slip motion along a prominent intracontinental fault, the Gastre Fault^32^ (Fig. 1c). The action and existence of this fault in Jurassic times have not been proved in the field^33^, opening to question the large block rotations and the basin’s role in accommodating them. Understanding the structure and growth of the Falkland Plateau Basin in increased detail is thus crucial for evaluating the correlation-based block motions of South Georgia, the Falkland Islands, and the Ellsworth Mountains.

Outcrop, drill-core, and dredge haul evidence from the Falkland Plateau Basin’s eastern, western and northern margins reveal the presence of Precambrian continental basement^34,35^. In the basin interior, however, reconnaissance-scale geophysical data were long unable to distinguish between the presence of extended continental crust underlain by a thick gabbroic underplate, and unusually thick oceanic crust^36–38^. Recently, high-resolution seafloor seismic refraction data along the basin’s central parallel revealed the presence of 10–12 km-thick crust of two layers underlying a thick infill between a pair of continent–ocean transition zones at the eastern and western margins^39,40^. The crystalline crust is devoid of the prominent reflections and very high velocities that tend to characterize underplated gabbro layers elsewhere in the world. The basin interior is thus now well-constrained as a product of igneous crustal growth. Despite this, the plate tectonic context of this growth and the basin’s later interactions with the growing Scotia Sea, and with them understanding of and confidence in the correlation-based rotations and translations of South Georgia, the Ellsworth Mountains, and Falkland Islands, remain unproved. One of the main reasons for this is the lack of reliable magnetic anomaly coverage of the basin, which could serve to identify the shapes and ages of magnetic reversal isochrons in its igneous floor.

### The AIRLAFONIA survey

**Figure 2:**
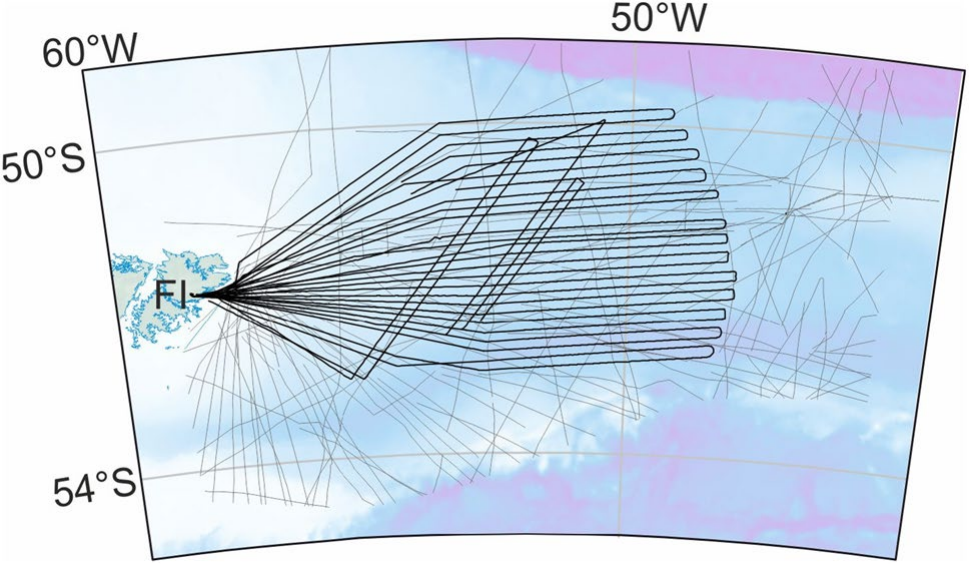
AIRLAFONIA flight tracks returning magnetic anomaly data (heavy lines) over the Falkland Plateau Basin. Legacy ship and helicopter tracks are shown as lighter lines. Background image: present-day bathymetry^41^. Map: Generic Mapping Tools^20^.

**Figure 3:**
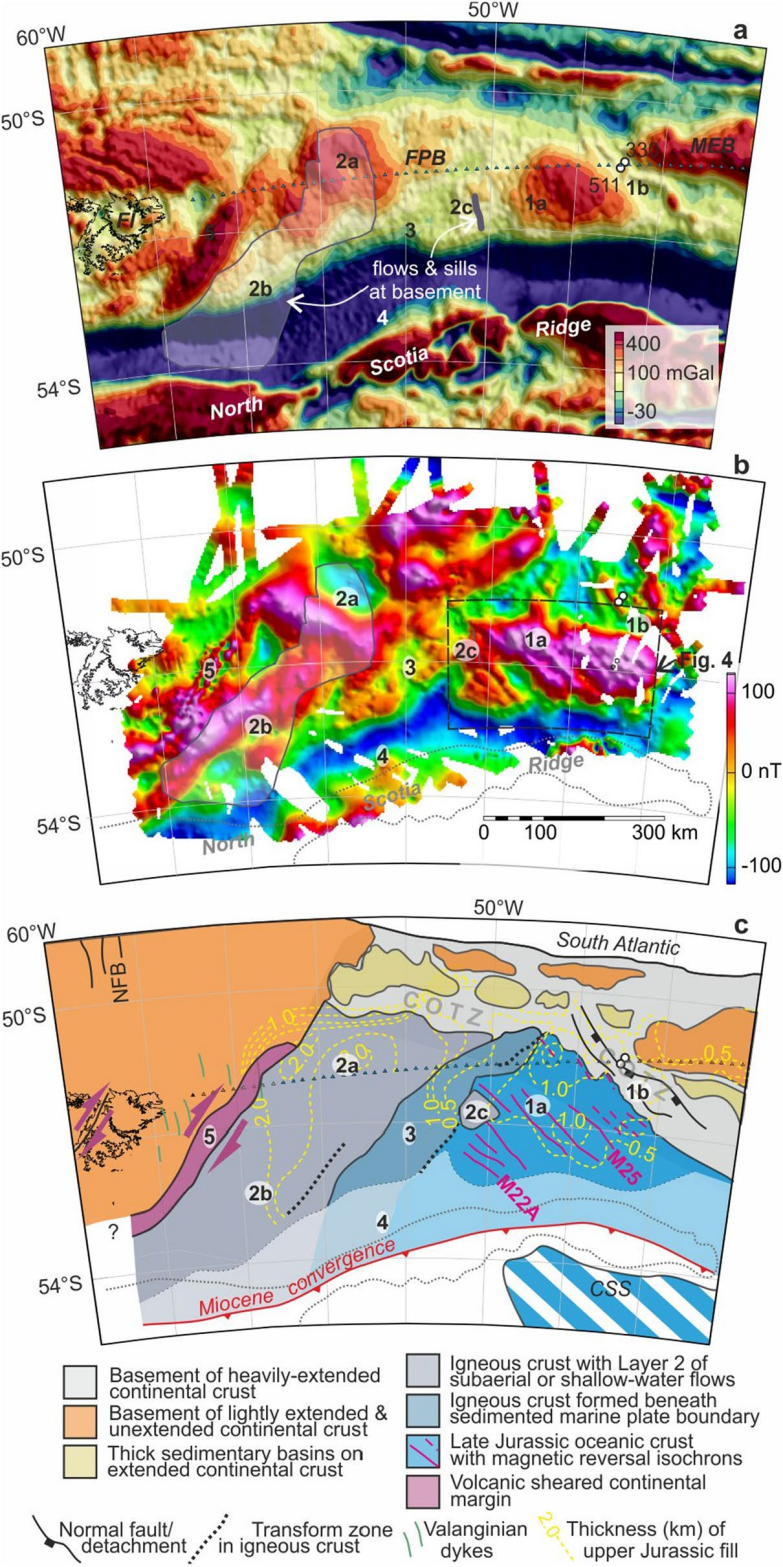
Potential field data and interpretation of the Falkland Plateau Basin. (**a**) Satellite-derived free-air gravity anomalies^42^. *FI* Falkland Islands, *FPB* Falkland Plateau Basin; *MEB* Maurice Ewing Bank. White disks: DSDP drill sites 330 and 511. 1a, 1b, etc.: Areas referred to in the text. Grey line and light grey transparency: seismically-imaged sills and lava flows at or in basement^31,34^. Triangles: seismic refraction profile^39,40^. (**b**) New total field magnetic anomaly grid (see methods). Box shows the area of magnetic reversal isochrons presented in more detail in Fig. 4. (**c**) Interpretations. *COTZ* continent–ocean transition zone. North trending Valanginian dykes and North Falkland Basin (NFB) faults are related to South Atlantic opening^26,31^. M22A, M25: interpreted Kimmeridgian-aged magnetic reversal isochrons. Upper Jurassic sediment thicknesses from seismic reflection data^34^. CSS: pre-Neogene location of late Jurassic oceanic crust of the central Scotia Sea. Maps a and b were built using Geosoft Oasis Montaj (https://www.seequent.com/products-solutions/geosoft-oasis-montaj/). Map c: new artwork created for this manuscript.

To help understand the Falkland Plateau Basin more clearly, the Alfred Wegener Institute flew AIRLAFONIA, an aerogeophysical survey of its interior, in November 2017 and November 2018. Both parts of the survey were flown with *Polar 6*, one of the Institute’s two Basler BT-67 aircraft, which are conversions of former Douglas C-47 Skytrain airframes. Magnetic intensity data were collected with a Scintrex Cs-3 caesium vapour magnetometer carried in a tail stinger. The data were compensated for aircraft effects in real time using the measurements of a three component fluxgate magnetometer mounted in the tail fin. Gravity data were gathered using a Gravimetric Technology GT2A gravity meter system, and tied to the International Standard Gravity Network via the absolute measurement point in Stanley, Falkland Islands, using measurements made with a portable LaCoste and Romberg gravity meter. AIRLAFONIA returned 25,185 line-kilometers of magnetic data along east–west and northeast-southwest oriented tracks designed to maximize coverage of the central part of the basin (Fig. 2). The majority of the flight tracks over the Falkland Plateau Basin were completed in level flight at 2000 ft above the sea surface, with occasional segments at 1000 ft or 3000 ft to avoid icing risk in heavy cloud. The east–west tracks were spaced at ~ 12 km to aid the generation of a continuous grid with resolution suitable for the identification of magnetic reversal isochrons. The data were cleaned of spikes and diurnal variations using the readings of a temporary stationary magnetometer on East Falkland. A continuous grid was built using standard levelling techniques applied to the airborne data within Geosoft’s *Oasis Montaj* geophysical processing and analysis environment, and then extended towards the eastern and northern margins of the basin by levelling a further 39,000 km of marine and helicopter legacy data to the AIRLAFONIA dataset (Fig. 3). Given the relatively small vertical separation of the marine and airborne data sets, and in order to retain detail, no vertical continuation was applied during levelling.

## Results

### Structure of the Falkland Plateau Basin and its margins

We interpret the tectonic evolution of the Falkland Plateau Basin with reference to five distinct areas of contrasting variability in our new magnetic anomaly grid, together with published free-air gravity anomalies derived from satellite radar altimeter data (Fig. 3a,b)^42^. In Area 1a, southwest of Maurice Ewing Bank in the eastern half of the basin, the new magnetic anomaly data reveal a set of five narrow linear northwest-trending magnetic anomalies. The anomalies coincide with a regional gravity high (~ 300 mGal) whose breadth and smoothness do not support the presence of significant structural or topographic variability in the basement that might explain their presence. The association instead clearly reflects the production of oceanic crust at a mid-ocean ridge during a period of geomagnetic field polarity reversals. The magnetic reversal anomalies terminate in the west along a northeast-trending line that may express a fracture zone formed at a stationary offset feature, possibly a transform fault, on the mid-ocean ridge crest during its lifetime.

Area 1b lies between the magnetic reversal anomalies and a single prominent positive magnetic anomaly lineation that coincides with a sinuous linear gravity high over gently southwest-dipping basement topography just 30 km seawards of the continental basement cored at DSDP Site 330^34,35,40^. Basement seismic velocity profiles indicate that extended continental crust continues in the basement between Site 330 and the gravity high^40^. The extended continental crust shows no evidence for large-scale extension-related magmatism in the form of seaward dipping reflectors in multiple seismic reflection profiles^34,43,44^, or high-velocity lower crustal bodies in seismic refraction data^40^. This combination of characteristics is typical of a magma-poor continent–ocean transition zone^45^. The transition zone runs WNW along the southern margin of Maurice Ewing Bank and, beyond it, appears to be transected by the Falkland Escarpment, the South American wall of a Cretaceous-aged transform fault in the South Atlantic. The excised northern parts behind the African wall lie in the Outeniqua Basin (Fig. 1a), south of South Africa^46,47^.

The smooth gravity anomaly field continues into the western part of the basin interior. Here, in Area 2a, northwest-striking magnetic anomalies are considerably broader than those in the east (Fig. 3b). The continuity of anomaly strike suggests that these magnetic anomalies express the action and products of a plate boundary with the same orientation as the mid-ocean ridge in the east. Area 2b, further south, shows northeast-trending features, also with long wavelengths. The broad anomalies of areas 2a and 2b coincide with a large province of flat to gently-dipping reflections from the deepest sedimentary fill of the basin, the top surface of the seismic basement, and within the uppermost basement, all of which have previously been related to the presence of nearly flat-lying sills and lava flows^31,34^. These sills and flows comprise the upper igneous layer of the basin’s three-layered crust^39,40^ and thus most likely express its growth by magma transport to and subsequent flow at or near the surface. Flows like this are observable at the present day along the active divergent plate boundary in the Afar Triangle^48^. By analogy to that region, the northwest-striking plate boundary in the western half of the Falkland Plateau Basin was likely to have been divergent, and to have occupied a subaerial or shallow marine basin. Area 2c marks a small further area with a smooth magnetic anomaly field and low-angle basement seismic reflections that is likely to have formed in a similar setting further east. The change in dominant anomaly strike between areas 2a and 2b may reflect an increase in along-strike segmentation of the boundary with time.

The transition between the shallow-to-subaerial divergent plate boundary in the west and the mid-ocean ridge in the east is marked by a ~ 100 km wide strip of spatially-disorganised low-amplitude magnetic anomalies, Area 3, across which the basin’s oldest sedimentary fill package thins eastwards^34^. We interpret these observations in terms of an eastwards-plunging segment, or eastwards-downstepping segments, of the divergent plate boundary whose igneous activity occurred within and beneath a wedge of marine sediments, where intense hydrothermal alteration suppressed the formation of strong magnetic susceptibilities in the igneous rocks^49^.

The Falkland Plateau Basin gives way southwards to the Scotia Sea via a chain of bathymetric and gravity highs known as the North Scotia Ridge (Fig. 3). The basin floor plunges southwards beneath a thick deformed sediment pile that makes up the ridge^50^, in the process forming a deep bathymetric trough. Magnetic anomalies over the trough and northern flank of the North Scotia Ridge (Area 4) are broad and subdued, consistent with increasing depth to the plunging basement, but otherwise retain a combination of northwest and northeast trends like those further north in the basin. These features are all consistent with downwards flexure of the basin floor in response to a history of near-orthogonal Miocene plate convergence, followed since Pliocene times by slight left-lateral shearing^4,50–52^. The new data reveal no additional evidence in the trough for the products of a system of Eocene and younger east-striking strike-slip faults that would be required to have accommodated South Georgia’s 1600 km of eastwards translation along the southern edge of the basin^11^. Together with the previous conclusions that there is also no evidence for such a system of faults further south in the floor of the Scotia Sea^4,11^, this tightly constrains the products of such a system, if it existed, to be deeply buried within the accretionary complex of the North Scotia Ridge.

### Age of the Falkland Plateau Basin

**Figure 4:**
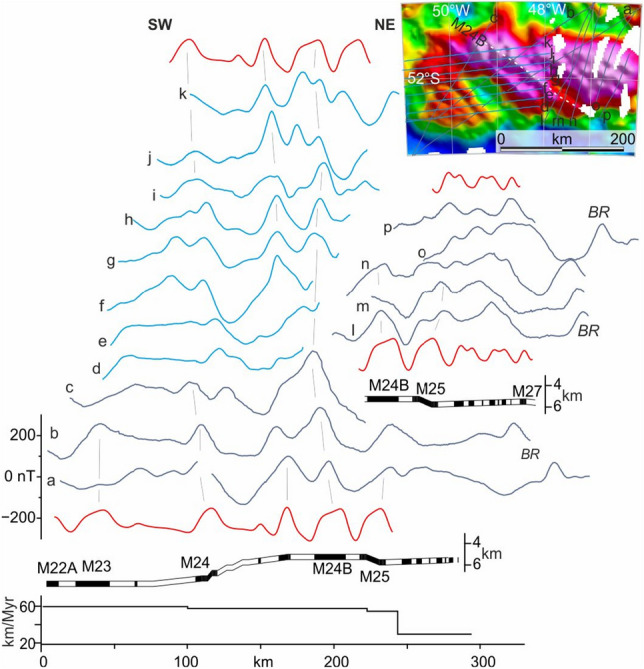
Synthetic magnetic anomaly model (red lines) of shipborne (dark blue) and AIRLAFONIA (light blue) profiles crossing linear reversal anomalies in Area 1a of the eastern Falkland Plateau Basin. All profiles projected along N45°E. Inset locates and names the profiles with respect to the magnetic anomaly grid of Fig. 3b; the map was built using Geosoft Oasis Montaj. The profile data are detrended by applying a 90 km-long high-pass filter. BR: anomaly over basement ridge in the continent–ocean transition zone west of Maurice Ewing Bank^40^.

The magnetic anomaly evidence for southwest-oriented plate divergence in the Falkland Plateau Basin terminates off the Falkland Islands at the basin’s southwest-striking continental margin (Area 5; Fig. 3). The strong narrow positive magnetic anomalies and narrow (~ 80 km) continental necking zone^39,53^ at this margin are consistent with it having hosted a steep volcanically-active fault zone along which the divergent relative plate motion evident from the basin interior was transformed into right-lateral lithospheric shearing. Onshore in the Falkland Islands, right-lateral transpression is also evident from structural studies^54^ and microtextural analyses of southwest-striking dolerite dykes^27^. Anisotropy of the dyke rocks’ magnetic susceptibilities further suggests that the shearing occurred whilst they cooled after being intruded^27^. Intrusion is dated to 182 Ma^55,56^, and thus places a Toarcian^57^ upper bound on the timing of transform motion at the margin of, and plate divergence in, the Falkland Plateau Basin. Correlation of cored sediments from DSDP sites 330 and 511 to unfaulted near-basement regional reflectors in the basin interior constrains the latest possible onset of seafloor spreading in response to plate divergence to a period during Tithonian–Callovian times (145–166 Ma)^43,44,58^. Under these constraints, a reasonable model likeness to the seafloor spreading anomalies can be achieved for the magnetic reversal isochron sequence M25–M22A (156–152 Ma^57^/Kimmeridgian; Fig. 4). A short sequence of relatively incoherent linear magnetic anomalies lying to the northeast of the modelled anomalies in the Falkland Plateau Basin might express the presence of pre-156 Ma oceanic crust (Fig. 4). The model divergence rate is fast (up to 60 km/Myr half-rate), but not unknown when compared to seafloor spreading in modern settings with comparable short young ridges^59^, and consistent with P-wave velocity evidence for melting in response to rapid mantle upwelling^40^. An alternative set of correlations would be possible for slightly slower spreading during the period 178–171 Ma (Toarcian; not illustrated)^60^. We can rule out other middle and late Jurassic ages because their high-frequency magnetic reversals would require unfeasibly-fast half spreading rates (> 150 km/Myr) well in excess of the maximum known half rate (100 km/Myr) from the Miocene Cocos-Pacific plate boundary^61^. We favour the Kimmeridgian model for two reasons. First, it is more consistent with the ages of cored and seismically-imaged thermal subsidence strata in the basin. Second, the isochrons would mark northwestwards continuations of the more extensive set of Kimmeridgian to early Cretaceous-aged isochrons (M25-M1n) previously interpreted from the Central Scotia Sea, which would have lain immediately to the southeast of the Falkland Plateau Basin until the Cenozoic opening of Drake Passage (Fig. 3c)^23^.

### A newly-recognized plate and its motion

**Figure 5:**
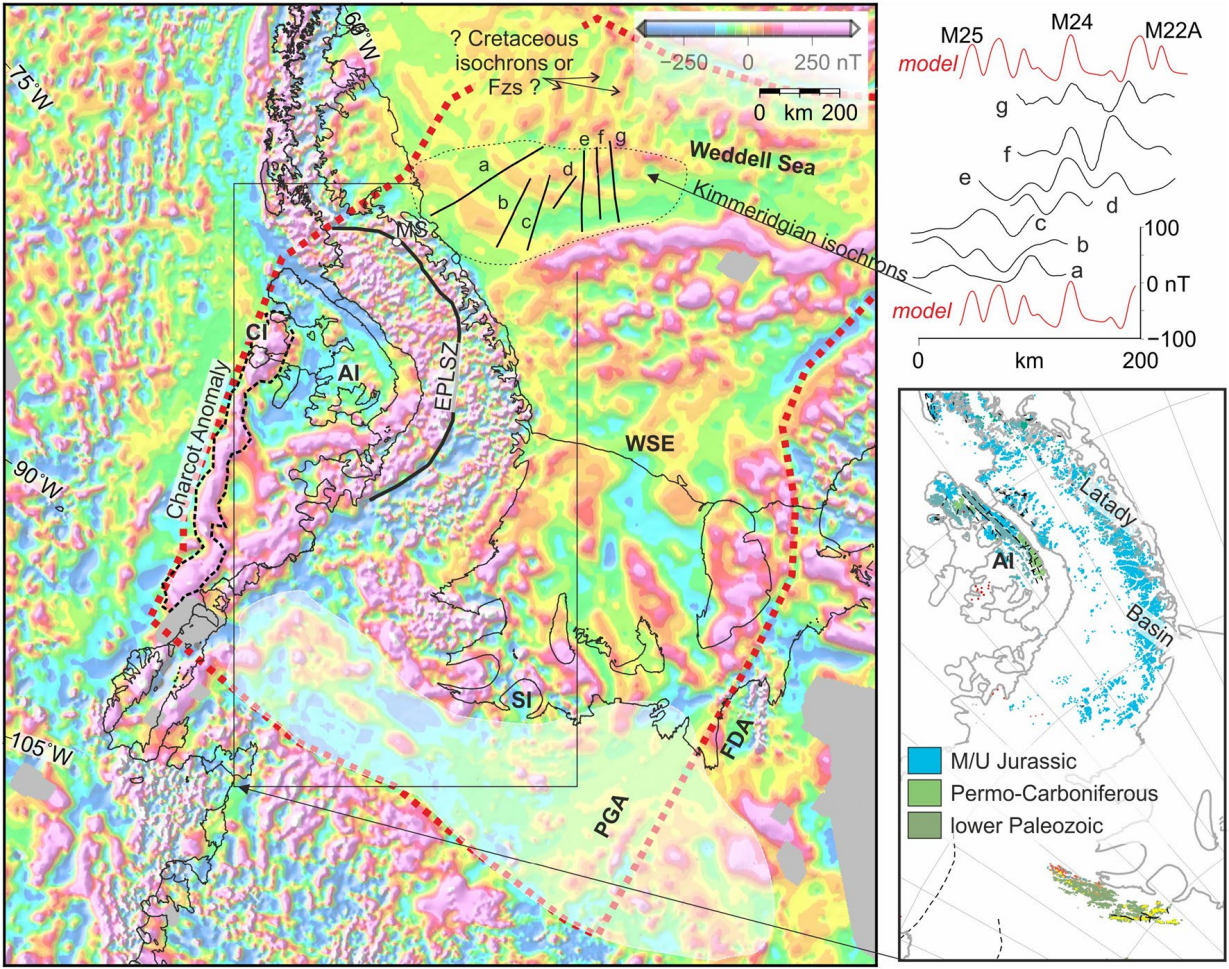
Antarctic constraints on motion of the Skytrain plate. Left: ADMAP2 gridded total field magnetic anomalies^65^ and some interpretations of them. Thick dashed red line surrounds the area interpreted to have been contained within the Skytrain Plate or affected by relative motions at any of its margins: *AI* Alexander Island, *CI* Charcot Island, *FDA* Forrestal and Dufek anomalies; *MS* Mount Sullivan, *PGA* Pagano Granite Anomaly, *SI* Skytrain Ice Rise, *WSE* Weddell Sea Embayment. Semi-transparent fill: area of subdued magnetic anomaly field west of the Ellsworth Mountains. Map: Generic Mapping Tools^20^. Upper-right inset: Profiles (**a**–**g**) over the older, more southerly, set of northwest-trending anomalies are modellable at low resolution using the same rates and timing of seafloor spreading as used in Fig. 4. Lower right inset: distribution of sedimentary rocks in the Palmer Land, Alexander Island and Ellsworth Mountains region (Data via https://data.gns.cri.nz/ata_geomap/index.html?). Map: QGIS v 3.14 (https://qgis.org/en/site/forusers/download.html).

Outside the Falkland Plateau Basin, Kimmeridgian times also saw the action of two further lengths of mid-ocean ridge. These ridges formed today’s Riiser-Larsen Sea and Mozambique and Somali basins. Together, they accommodated the divergence of two large plates that was splitting Gondwana into eastern (bearing Antarctica, India and Australia) and western (bearing South America and Africa) parts at half spreading rates of 20–25 km/Myr^62,63^. For the Falkland Plateau Basin to have opened along the same plate boundary, half rates implied by its magnetic reversal isochrons would therefore need to have been half as fast as modelled in Fig. 4^6,63^. The divergence rate discrepancy thus reveals the action of a third plate. The right-lateral sense of shear^27,54^ at and in the basin’s continental margin with the Falklands requires this plate to have lain on the southern flank of the divergent plate boundary, in what would have been the growing Weddell Sea. Starting in Kimmeridgian times, the plate boundary between the Falkland Plateau Basin and Weddell Sea was thus a divergent boundary between the West Gondwana plate in the north and a hitherto-unrecognized southern plate. We refer to this plate as ‘Skytrain’, after the Skytrain Ice Rise in its interior at the southern edge of the Weddell Sea Embayment (Fig. 5).

The AIRLAFONIA data show how, in the process of diverging from the Skytrain plate, West Gondwana acquired a length of extended continental margin in the north of the Falkland Plateau Basin. A prominent (100°) left-handed bend at the western end of this margin segment marks its transition to a sheared continental margin segment off East Falkland (Fig. 3c). The Skytrain-side conjugates to these margin segments are not immediately obvious further south. A conjugate to the Kimmeridgian-aged oceanic crust of the Falkland Plateau basin, in contrast, is easier to identify in the form of a package of northwest-trending magnetic anomaly lineations in the southwesternmost Weddell Sea (Fig. 5). These anomalies are raised over sources situated in deep (~ 10 km below sea level) basement rocks and sampled on widely-spaced and variably-oriented aeromagnetic profiles^64,65^. Despite their low resolution, they are unanimously interpreted as signatures of magnetic reversals during the growth of oceanic crust and, based on the age of exposed post-rift strata in the neighbouring Latady Basin, most likely date from Kimmeridgian times^64,66,67^. With their low resolution as a caveat, they can be modelled as a set of Kimmeridgian isochrons similar to that in the Falkland Plateau Basin (Fig. 5). Just to the north, in the western Weddell Sea, a set of NNE-trending anomalies is more controversial, interpreted either as the signals of magnetic susceptibility contrasts at fracture zones^64^, or as the record of a short-lived episode of east-directed early Cretaceous (~ 138–135 Ma) plate divergence^66^.

**Figure 6:**
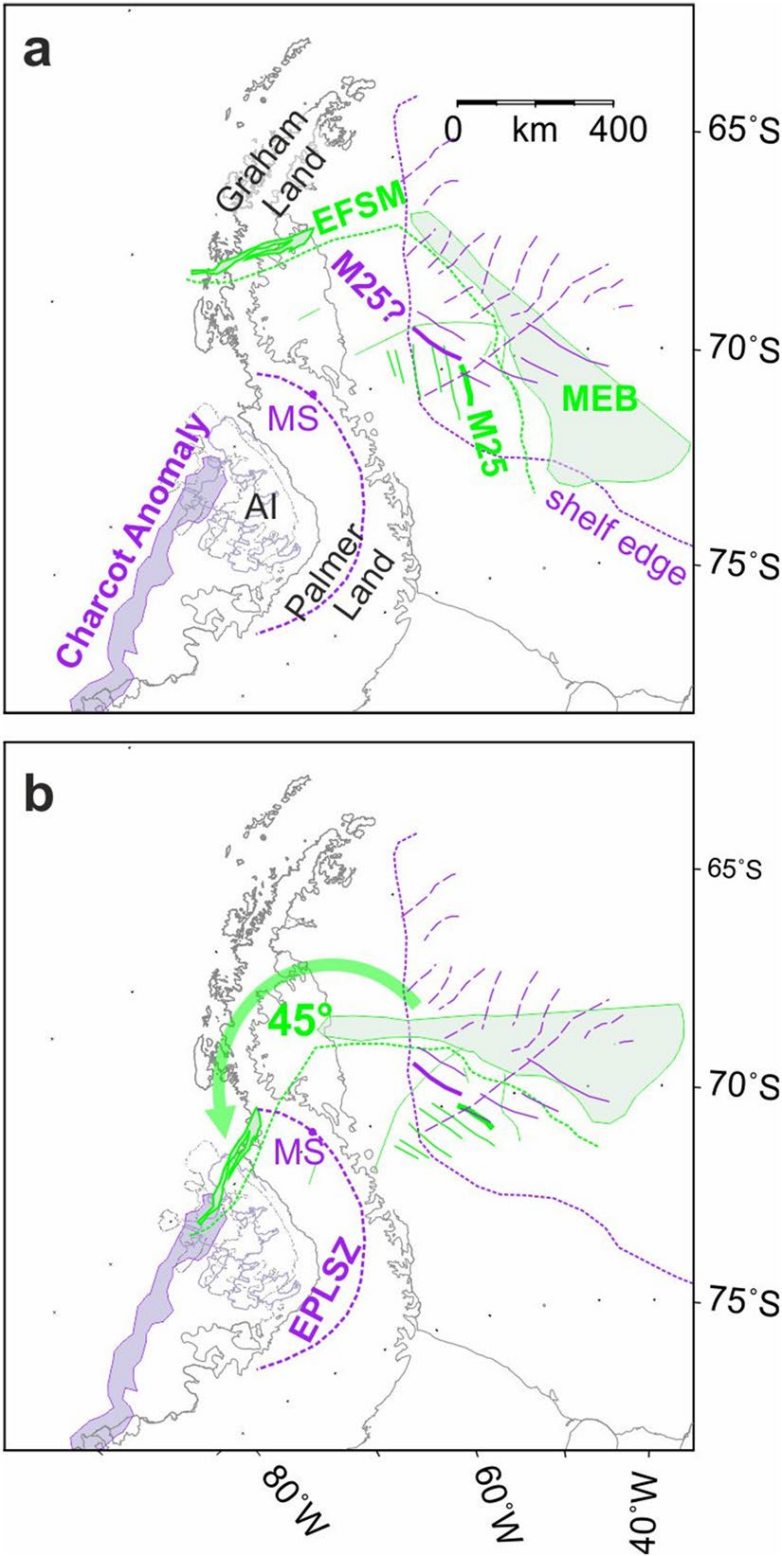
Coarse corrections to a previous regional reconstruction for newly-recognized effects of Skytrain plate motion. (**a**) The extended continental margin segment of Maurice Ewing Bank (MEB) and neighbouring East Falkland sheared margin segment (EFSM), and Kimmeridgian magnetic reversal isochrons of the Falkland Plateau (all in green, M25 labelled) are rotated using a full-fit rotation based on a previous^3^ study of seafloor spreading data from the Weddell Sea. (**b**) Effects of correcting for the 45° misfit between the Falkland Plateau Basin isochrons and their conjugates in the southwestern Weddell Sea (purple lines). Alexander Island and the Charcot Anomaly are shown in their locations prior to Neogene opening of George VI Sound. *AI* Alexander Island; *EPLSZ* Eastern Palmer Land Shear Zone; *MS* Mount Sullivan. Rotations completed using authors’ own software. Maps: Generic Mapping Tools^20^.

AIRLAFONIA thus delivers important new constraints for plate kinematic reconstructions in southwestern Gondwana: a distinctive pair of extended and sheared continental margin segments in the northern Falkland Plateau Basin, the location of conjugates to the set of magnetic isochrons previously known from the southwest Weddell Sea, and their Kimmeridgian date. Figure 6a shows a reconstruction of the new Falkland Plateau constraints into the Weddell Sea made using a recent set of rotation parameters that predate recognition of the Skytrain plate^3^. The sets of conjugate Kimmeridgian anomalies misalign by more than 45°. Figure 6b realigns the anomalies, coarsely correcting for much of the effect of the Skytrain plate’s motion and allowing for a clearer understanding of the conjugates to the continental margins of the Falkland Plateau Basin. The correction aligns the Falkland Plateau Basin’s sheared continental margin segment with the Charcot Anomaly, a prominent magnetic lineament that runs for 700 km parallel to the continental shelf west of Alexander Island (Fig. 5). This shelf was most recently tectonically active as a subduction-affected continental margin in Paleogene and later times. The Charcot Anomaly is regarded as enigmatic in this context because of the lack of extensive Paleogene and younger volcanic and plutonic rocks^68^. Instead, on Charcot Island, which the anomaly crosses, deformed sedimentary rocks mark a continuation of the Permo-Carboniferous LeMay Group of Alexander Island (Fig. 5)^69,70^. On Alexander Island itself, these rocks are unconformably overlain by thick upper Jurassic sediments of the Fossil Bluff Group (Fig. 5)^65^, which were locally intruded by basaltic and rhyolitic lavas around the time of deposition^71^. These relationships are the same as would be expected of a conjugate sheared continental margin to that off East Falkland, at which the Permo-Carboniferous Lafonia Group can be traced offshore beneath the late Jurassic sedimentary fill of the Falkland Plateau Basin with its sills and lava flows^31,55,72^. The source of the Charcot Anomaly and its conjugate offshore East Falkland may therefore be doleritic rocks intruded into and erupted at a magmatically-active late Jurassic transform plate boundary.

In Fig. 6b, rotation of the extended continental margin of the northern Falkland Plateau Basin aligns it parallel to the continental shelf edge in the southwestern Weddell Sea, and its westwards projection along the northern stretch of the Eastern Palmer Land Shear Zone. Here, the shear zone runs parallel to the sharp neck in the Antarctic Peninsula that marks the boundary of its broad southern segment, Palmer Land, with the narrower northern Graham Land segment. Exposed at Mount Sullivan (Figs. 5, 6), this part of the Eastern Palmer Land Shear Zone is a major early-^73^ or mid^74^-Cretaceous convergent shear zone whose action produced a set of mylonitic deformation zones in an early Jurassic extension-related granitoid pluton^75,76^. These relationships support an interpretation of the northern section of the shear zone as marking a tectonically-inverted segment of pre-Kimmeridgian extended continental margin on the Skytrain plate.

**Figure 7:**
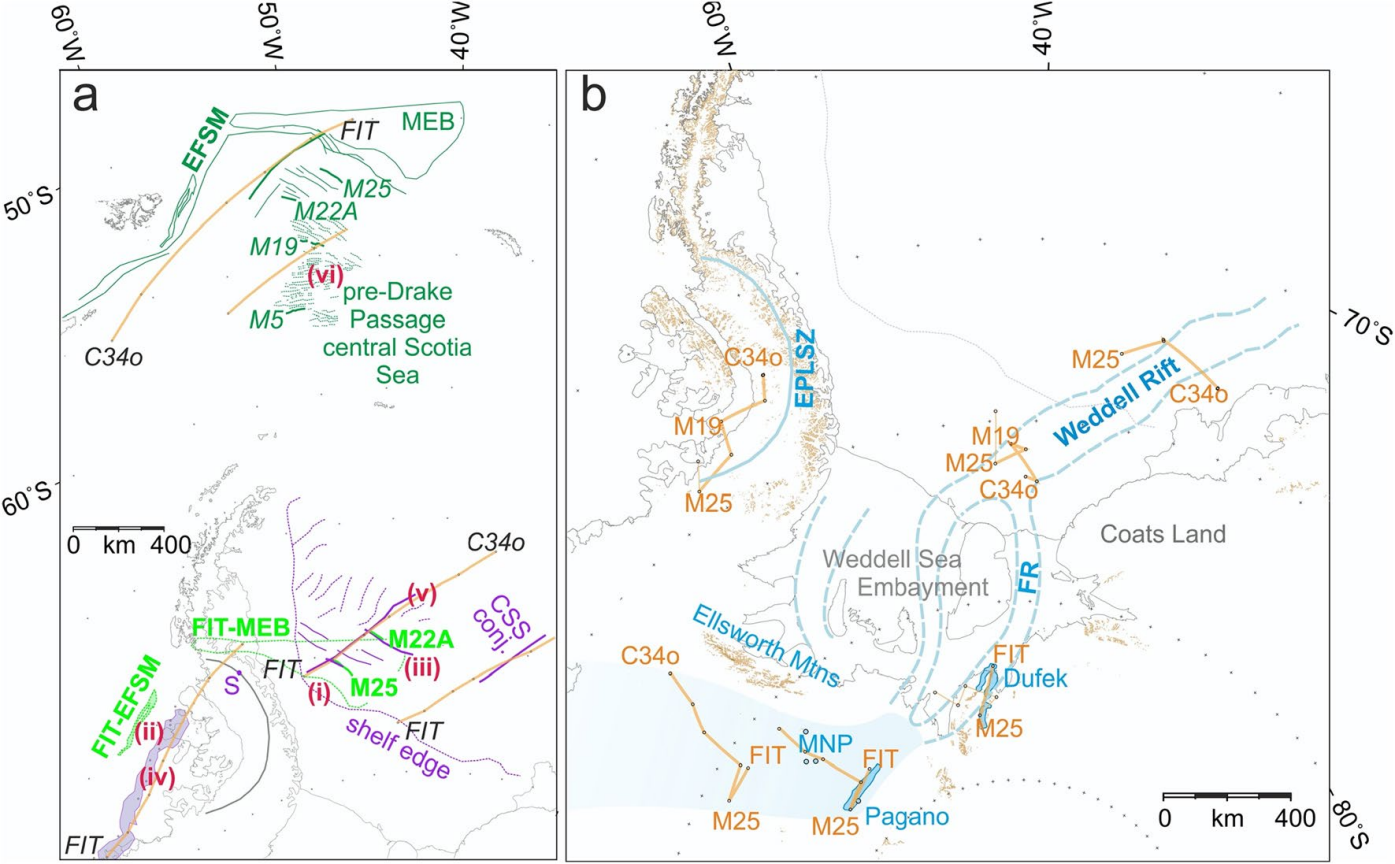
(**a**) Joint flowline-isochron visual-fit modelling for Skytrain-West Gondwana plate motion. (i)–(vi) refer to six sources of constraint used for visual fit modelling, as described in the text. West Gondwana-side constraints from the Falkland Plateau Basin and pre-Drake Passage-located central Scotia Sea are shown in dark green (*EFSM* East Falkland sheared margin segment, *MEB* Maurice Ewing Bank). Light green features in the Weddell Sea and Palmer Land are Falkland Plateau constraints after rotation by the labelled parameters (cf. Table 1) into coincidence with their Skytrain-side targets. Skytrain-side constraints from the Weddell Sea and Palmer Land (see also Figs. 5 and 6) are shown in purple. S: Mount Sullivan, in the Eastern Palmer Land Shear Zone. Orange lines are model flowline segments that track the motion of fixed points on the Skytrain-West Gondwana divergent plate boundary in the period until C34o (126 Ma). CSS conj.: approximate area of the southern Weddell Sea expected to host the conjugate seafloor to the central Scotia Sea. (**b**) Model motion of points on the Skytrain-East Gondwana plate boundary with respect to the interior of the Skytrain plate during mid-Jurassic and early Cretaceous times. Brown areas: outcrop. Blue dashed lines: margins of interpreted rifts in the Weddell Sea Embayment^80,81^, *FR* Filchner Rift. Heavy blue fill: magnetic anomalies associated with Dufek Massif and Pagano Nunatak intrusions. Light blue fill: area of subdued magnetic response (cf. Figure 5). All rotations completed using authors’ own software. Maps: Generic Mapping Tools^20^.

Based on the interpretation of Fig. 6b, the Skytrain plate’s motion with respect to West Gondwana seems to have shaped the growth of the Falkland Plateau Basin, central Scotia Sea, southern Weddell Sea, and an area of extended continental margin that once lay north of the Eastern Palmer Land Shear Zone but has been tectonically shortened. Figure 7 describes our use of the new constraints from these areas within a model for motion of the Skytrain plate relative to the West Gondwana plate. The model is built by searching for a set of rotation poles that provide simultaneous visually-acceptable fits of (i) the conjugate extended continental margin segments (Figs. 3 and 5) to one another, (ii) the conjugate sheared continental margin segments (Figs. 3 and 5) to one another, (iii) the conjugate Kimmeridgian magnetic isochrons (Figs. 3 and 5) to one another, (iv) the orientations of the sheared margin segments to synthetic flowline segments drawn along small circles about the rotation poles, (v) the orientations and lengths of fracture zone traces, interpreted from gravity and magnetic anomalies, in the floors of the Falkland Plateau Basin (Fig. 3) and southern Weddell Sea (Fig. 5^42,64^) to synthetic flowline segments, and (vi) the spacing of magnetic reversal isochrons M19 and M5^23^ in the central Scotia Sea, with their locations adjusted for the opening of Drake Passage^4^. The greater (by > 4 km) depth to source and very sparse coverage of reliable magnetic track lines in the southern Weddell Sea^64,65^ left us unable to identify a detailed set of conjugate magnetic reversal isochrons to those of the central Scotia Sea for the modelling. Table 1 lists the model rotations together with a set of Skytrain–East Gondwana rotations calculated for mutually-constrained chrons by addition in the regional plate circuit^6,63,77–79^.

**Table 1:** Euler finite rotation parameters for reconstruction of the Skytrain plate with respect to the west Gondwana (WGON) and East Gondwana (EGON) plates.

Chron	Age, Ma^57^	Latitude	Longitude	Angle	Target plate	Method
C34o	126	72.6°S	90.1°W	47.9°	WGON(SAM)	Circuit closed without Skytrain plate^79^
M5	131	69.0°S	94.7°W	44.3°	WGON(SAM)	See Fig. 7
M19	146	51.6°S	112.6°W	27.6°	WGON	See Fig. 7
M22A	152	37.6°S	118.7°W	22.6°	WGON	See Fig. 7
M25	156	19.4°S	121.3°W	20.7°	WGON	See Fig. 7
FIT	~ 180	4.6°S	121.4°W	20.6°	WGON	See Fig. 7
C34o	126	73.0°N	149.0°E	0.0°	EGON	Circuit closed without Skytrain plate^77−79^
M5	131	73.0°N	149.0°E	7.9°	EGON	Circuit completion with Skytrain^77−79^
M19	146	78.7°N	117.8°E	31.5°	EGON	Circuit completion with Skytrain ^63,77,78^
M25	156	79.8°N	137.3°E	45.8°	EGON	Circuit completion with Skytrain ^63,77,78^
FIT	~ 180	78.3°N	122.7°E	53.2°	EGON	Circuit completion with Skytrain ^63,77,78^

The end of the Skytrain plate’s independent motion would have seen the mid-ocean ridge in the Weddell Sea begin to accommodate divergence of the South American/West Gondwana plate from the East Antarctic/East Gondwana plate. Explicit closure of the regional plate circuit shows that this was certainly the case by 84 Ma^82^. A lower-resolution test has shown the circuit may already have been closed at 126 Ma (chron C34o)^3^. This earlier date is also consistent with the absence of evidence for a mid-to-late Cretaceous aged East Gondwana-Skytrain plate boundary transecting the oceanic lithosphere of the Weddell Sea. Based on this, we force our regional plate model circuit to close without any Skytrain plate motion beginning at 126 Ma (Table 1), and so restrict our interpretation of the plate’s role in regional tectonics to early Cretaceous and Jurassic times.

The model for Skytrain-West Gondwana motion features relatively smooth northeast-southwest oriented plate divergence (Fig. 7a). Its largest uncertainty is in the stage between chrons M22A and M19, over which its constraints change from two-sided fitting of conjugate isochrons in the Weddell Sea and Falkland Plateau Basin to a one-sided seafloor spreading record in the central Scotia Sea whose original location and orientation within the West Gondwana plate we can only coarsely estimate based on a model for the opening of Drake Passage^4^. This uncertainty propagates into the Skytrain-East Gondwana model, to which the M19 rotation imparts prominent bends and reversals that may consequently be artefactual. The distinct bend at chron M25, in contrast, is a robust feature because it sums into the model from clearly-defined bends in fracture zone orientations in the Southwest Indian Ocean^63^. Prior to M25, motion of the East Gondwana plate with respect to the Skytrain plate is directed close to south in the east of the Weddell Sea Embayment, but towards the west in the region of the Ellsworth Mountains (Fig. 7b). After M25, despite the uncertainty stemming from the estimated location and orientation of the central Scotia Sea prior to Drake Passage opening, it is clear that Skytrain-East Gondwana motion was defined by stage poles that were located near Coats Land. The field of relative motion accommodated within the East Gondwana and Skytrain plates’ shared boundaries is strongly curved because of its proximity to those poles. It requires a spatially complex pattern of accommodation. Broadly, this complexity is characterized by north–south oriented convergence in the area south and west of Coats Land and, further north, northeast-southwest oriented conservative and, later, southeast-northwest divergent motions.

The southern parts of the Skytrain plate’s boundaries with the West and East Gondwana plates would initially have been located in continental crust that was unusually thick and warm as a consequence of many millions of years of plate convergence at the paleo-Pacific margin of Gondwana. Strain would have accumulated slowly, as plate motion estimated from the rotations averages around 7 mm/yr. These conditions are likely to have manifested themselves in the development of broad and unstable plate boundary zones. This is consistent with the interpreted presence of a complex of buried rifts beneath the Weddell Sea Embayment^81^ (Fig. 7b). Further north and east, relative plate motion was faster and the lithosphere older and cooler. Here, the Skytrain plate’s boundaries were localized and generated the oceanic floor of what is now the southern Weddell Sea.

### Regional tectonic history with the Skytrain plate

**Figure 8:**
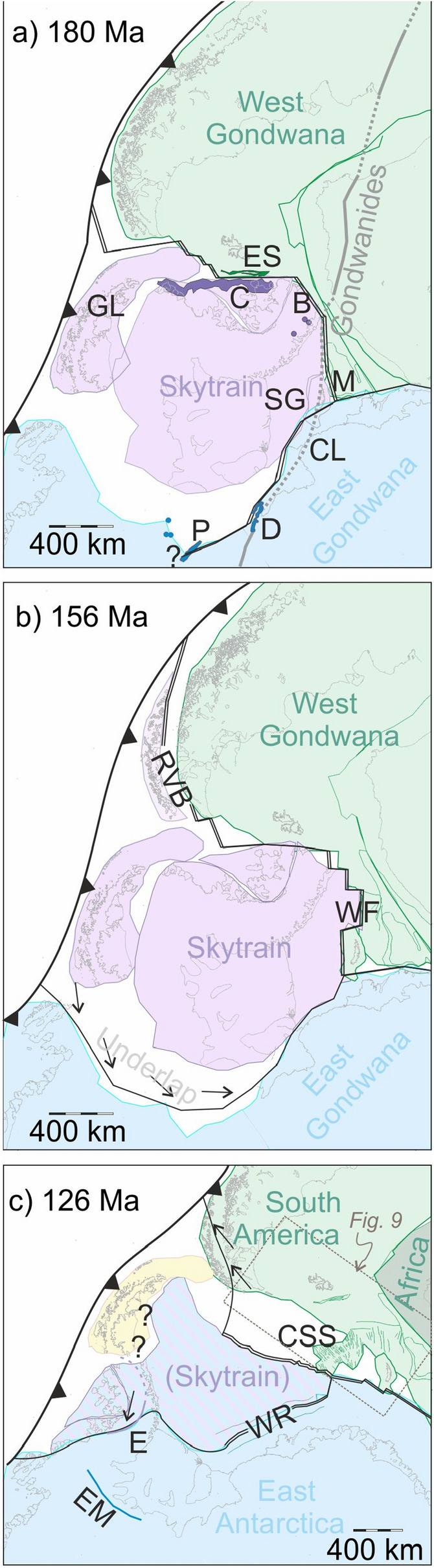
Regional tectonic reconstructions featuring the Skytrain plate at (**a**) its onset around 180 Ma (FIT), (**b**) 156 Ma (M25), and (**c**) its incorporation into the East Antarctic plate around 126 Ma (C34o). *B* back-arc basalts of northern Latady Basin; *C* Charcot Anomaly; *CL* Coats Land; *CSS* central Scotia Sea; *D* Dufek Massif; *E* southern part of Eastern Palmer Land Shear Zone; *EM* Ellsworth Mountains; *ES* East Falkland sheared margin magnetic anomaly; *GL* Graham Land; *M* Maurice Ewing Bank; *P* Pagano granite; *RVB* Rocas Verdes Basin; *SG* South Georgia; *WF* southern Weddell Sea and Falkland Plateau basins; *WR* Weddell Rift. Toothed lines: subduction zones; double lines: mid-ocean ridges and continental extensional basins; single lines with arrows: collision zones, unadorned single lines: transform plate boundaries. Rotations completed using authors’ own software. Maps: Generic Mapping Tools^20^. All plate outlines used are included as ASCII files in Supplement 1.

Figure 8a shows a regional reconstruction to the early Jurassic time (~ 180 Ma) when the Skytrain plate started to move independently. It is built using the “FIT” rotation parameters of Table 1. The reconstruction presents the Charcot and East Falkland sheared margin segments, and the extended margin segments of southern Maurice Ewing Bank and southwestern Weddell Sea as near-fitting conjugates. South Georgia is shown as a conjugate to part of the Weddell Sea margin a little further east, requiring it after 180 Ma to have spent a short period on the Skytrain plate, in order to open the present-day north–south gap between it and Maurice Ewing Bank. The early Jurassic location of Graham Land is not directly constrained by the Skytrain plate concept. We have shown Graham Land in a location at the active margin of Gondwana and suggest that it may at this time have formed part of the Skytrain plate.

Intrusion of dykes^26,27,55^ into the right-lateral transform zone on the Falkland Islands, eruption of back-arc basalts^83^ into the northern Latady Basin, and intrusion of the Mount Sullivan and related granitoids^76^, date the onset of the Skytrain plate’s motion with respect to West Gondwana to around 182–177 Ma (Toarcian). Synchronous activity on the Skytrain-East Gondwana plate boundary may have accompanied the intrusion of gabbros at the Dufek Massif and Forrestal Range^84^, and S-type granites at the Martin, Nash and Pirrit Hills, and Pagano Nunatak^85^. Previous studies, using remote sensing data, have conflictingly interpreted mid-to-late Jurassic right lateral shearing at the Support Force Lineament neighbouring the Dufek intrusion^86^, but left-lateral shearing in a broad region north of Pagano Nunatak^87^. We note that the elongated magnetic anomalies associated with the Dufek gabbro and Pagano granite are both rotated 5–20° clockwise away from the direction of mid-Jurassic model shearing (Fig. 7b), consistent with their intrusion into gentle releasing bends in a right-lateral shear zone. The Skytrain-East Gondwana and Skytrain-West Gondwana boundaries would have met with the East Gondwana-West Gondwana plate boundary at a triple junction somewhere in what is now the northeastern Weddell Sea Embayment off Coats Land.

Figure 8b shows a late Jurassic reconstruction for the time around 156 Ma (chron M25). By this time, seafloor spreading was well established on the Skytrain-West Gondwana plate boundary in the southern Weddell Sea and Falkland Plateau Basin. This time is also around that of the onset of oceanic crustal accretion in the Rocas Verdes Basin, which is today known only from ophiolitic remnants in Tierra del Fuego and the southern Patagonian Andes^3,88^. The basin may have opened along a western reach of the Skytrain-West Gondwana plate boundary that was abandoned again by around 141 Ma^89^. A large underlap further south in the reconstruction illustrates the Skytrain-East Gondwana rotations’ requirement for post-156 Ma plate convergence in the region south and west of the stage pole in Coats Land. Near the Ellsworth Mountains, the timing and orientation of this convergence (Fig. 7b) is consistent with structural evidence for a post-Permian phase of oblique right-lateral collision that has previously been attributed to the Permian–Triassic Gondwanide Orogeny (Fig. 1)^7,14,28^. An alternative, or additional, attribution to much later convergence of the Skytrain and East Gondwana plates would also explain why low temperature geochronological data from the mountains reveal an episode of uplift and denudation that was underway at 141 Ma and continued well into the early Cretaceous, long after the end of extensional rifting at the margin of the Weddell Sea to which it was previously attributed^3,90^. In such a revised interpretation, the uplift would accompany crustal thickening and the development of a late Jurassic to early Cretaceous transpressional to obliquely-convergent fold and thrust belt. An enigmatic 250–350 km-wide zone of subdued magnetic anomalies over the region of thick ice west of the Ellsworth Mountains (Figs. 5, 7b)^65^ may express the thick non-volcanic upper crust comprised of this fold and thrust belt.

Figure 8c shows a reconstruction to early Cretaceous times around 126 Ma (chron C34o). We consider this to be the latest possible time for independent motion of the Skytrain plate. The plate boundaries it depicts around the Skytrain plate are in the process of being abandoned as sites of tectonic activity. With this, the fragment of oceanic crust in what is now the central Scotia Sea, which had previously accreted at the Skytrain-West Gondwana plate boundary, is in the process of being incorporated into those parts of the West Gondwana plate that were by now moving independently as the South American plate^23^. The underlap west of the Ellsworth Mountains is fully closed; collision there had ended. We note that the period and sense of closure in the western parts of the underlap both broadly match some interpretations of transpressional activity on the Eastern Palmer Land Shear Zone^73^. Based on this, we suggest tentatively in Fig. 7b that, at least in the southern part of the shear zone, transpression might have occurred within the Skytrain-East Gondwana plate boundary zone at a time when ongoing convergence had caused it to become very broad. This interpretation resolves recently-identified difficulties in reconciling paleomagnetic data from the Antarctic Peninsula with the notion of the Palmer Land Event as a product of later (mid-Cretaceous; 107–103 Ma) accretion of far-travelled terranes^91^. This does not rule out the possibility that the mid-Cretaceous dates might record a second, post-Skytrain, phase of activity on the Eastern Palmer Land Shear Zone during the amalgamation of Graham Land, which paleomagnetic data^92^ suggest is unlikely to have rotated as part of the Skytrain plate, with Palmer Land. In the southern Weddell Sea, the extensional eastern end of the East Gondwana-Skytrain plate boundary (Fig. 7b) has recently been abandoned to form the fossil Weddell Rift parallel to the Coats Land coast^80^.

## Discussion

### One plate and multiple orogens; not one orogen and multiple plates

The Skytrain plate’s late Jurassic to early Cretaceous collisional rotation, about a nearby Euler pole with respect to East Gondwana, gives rise to ~ 55° of anticlockwise rotation of features borne within the body of the plate. A coarse uncertainty estimate of ± 5° on this value can be made based on the range of acceptable visual alignments of the East Falkland and Charcot Island conjugate magnetic anomalies. In comparison, the Ellsworth Mountains’ Cambrian geomagnetic pole is rotated anticlockwise by 85 ± 16° (α95) away from its Gondwanan counterpart^19^. Within these combined uncertainties, up to ninety percent of the Ellsworth Mountains’ paleomagnetic rotation can be explained by Jurassic-early Cretaceous motion of the Skytrain plate. If the Skytrain plate concept is to be accepted in this sense, however, then it becomes necessary to revisit the virtual geomagnetic poles determined in two Jurassic granites from Pagano Nunatak and Nash Hills^18^, both of which lie only 15° away from their East Antarctic counterpart. This is also procedurally necessary in view of the fact that those poles were determined in the absence of paleo-horizontal control from the granites.

In contrast, the 120° of apparent polar wander determined on ~ 182 Ma dykes in the Falkland Islands is not consistent with a rigid plate rotation. It can instead be more plausibly understood as a signal of sub-plate-scale rock rotations occurring on a transform segment of the Skytrain–West Gondwana boundary within cooling dyke rocks undergoing active right-lateral shear deformation^27^ and/or, at slightly larger scale, between pairs of right-lateral strike-slip faults in the same boundary zone^93,94^. These interpretations confine the Falkland Islands to the western margin of the Falkland Plateau Basin during and prior to its opening, requiring their nearest regional correlative rocks to be located in Patagonia. Patagonia has long and repeatedly been suggested as an alternative to southern African for regionally correlating the geology of the Falkland Islands^24,25^.

The recognition of local late Jurassic and early Cretaceous-aged transpressional orogens at the margins of the Syktrain plate strongly questions whether it is necessary to regard the Falkland Islands and Ellsworth Mountains as displaced stretches of the Permian–Triassic Gondwanide orogenic belt, which lay considerably further inboard in Gondwana (Fig. 8a). The regional correlations on which reconstructions of the Gondwanide Orogen are predicated can be viewed not as a consequence of the orogen’s assumed original continuity or linearity, but instead less prescriptively and more robustly as a consequence of the prior existence of a set of large and stratigraphically very similar intracratonic basins within Gondwana^95^. Any pair of Mesozoic or younger fold belts affecting the interiors of one or more of these basins would be expected to expose correlatable Paleozoic stratigraphies, without the correlations defining the fold belts’ original relative locations at a level of precision much better than the basins’ diameters.

### South Georgia and the conjugate to the Weddell Sea

Quantitative plate kinematic reconstructions of the Scotia Sea can only explain around half of South Georgia’s proposed translation to its present location from a Jurassic back-arc basin in Tierra del Fuego^11,22,51^. This difficulty prompted a reinterpretation of South Georgia’s main stratigraphic elements, which are widespread in southwest Gondwana, as features of a Jurassic–early Cretaceous extended continental margin developed over a region of mantle left anomalously fertile by metasomatic alteration during earlier prolonged low-angle subduction^22^. Subsequently, however, U–Pb age distributions in detrital zircon populations for South Georgia and Tierra del Fuego were shown to display comparable middle Jurassic and late Albian peaks, which were taken to reassert the Mesozoic back-arc interpretation^9^.

**Figure 9:**
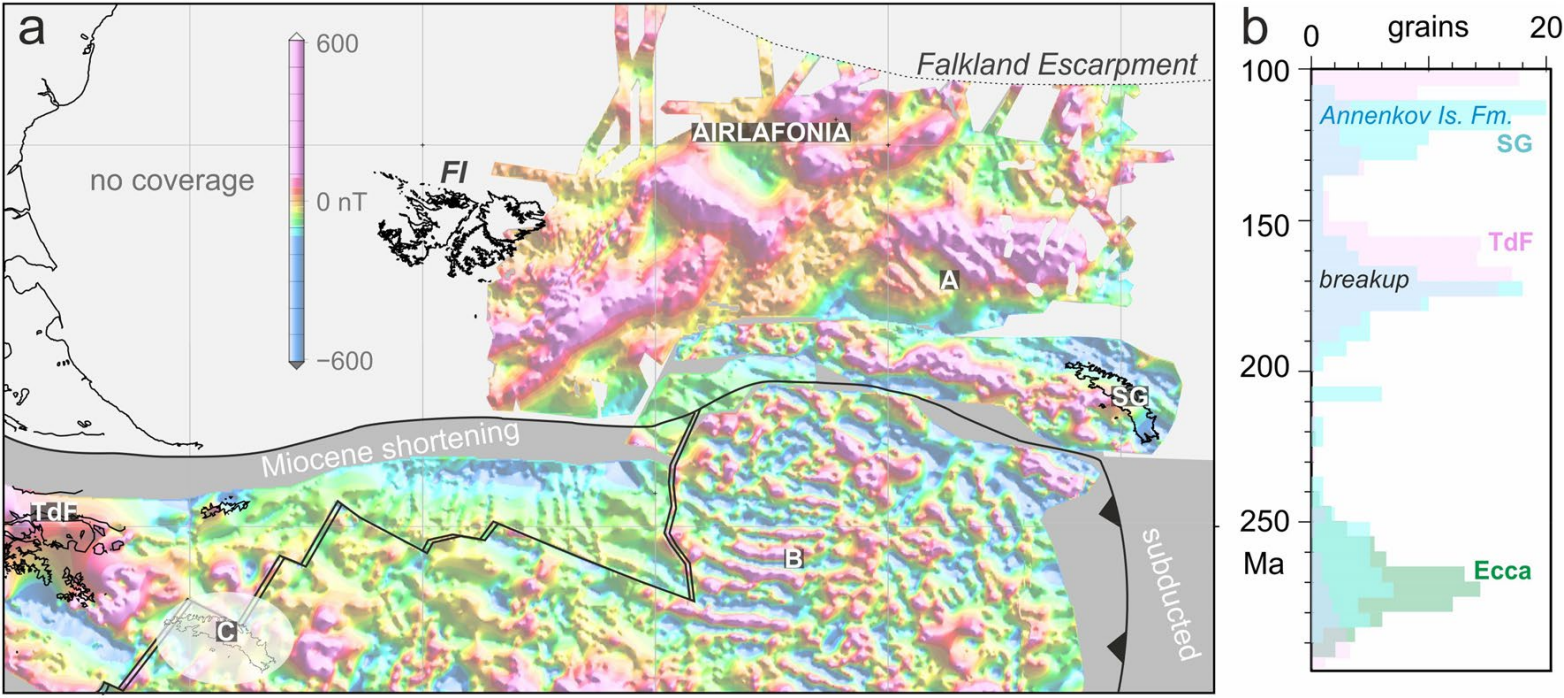
(**a**) Detailed reconstruction^4^ for a time prior to opening of the oceanic basins in the west and east Scotia Sea, made using magnetic anomaly grids from AIRLAFONIA (Fig. 3b) and from the Scotia Sea^51^. For the regional context, see Fig. 8c. Coastlines of South Georgia (SG), the Falkland Islands (FI), and present-day South America are included for orientation. The Kimmeridgian magnetic reversal isochrons in the Falkland Plateau Basin (A) are contiguous with a set of late Jurassic to early Cretaceous isochrons in the central Scotia Sea (B), forming a lithospheric barrier to eastwards translation of South Georgia from an alternative proposed location at this time (C), in a Mesozoic-aged back-arc basin off Tierra del Fuego (TdF). (**b**) U–Pb zircon age distributions from South Georgia^9^, Tierra del Fuego^96^, and the Ecca Formation^97^ in South Africa, which would have lain immediately north of the Falkland Escarpment prior to 140 Ma. Grid rotations and map: Generic Mapping Tools^20^.

Figure 9 shows a reconstruction of gridded magnetic anomalies to a time when the Mesozoic back-arc interpretation requires South Georgia not yet to have parted from Tierra del Fuego^9^ and the oceanic basins of the west Scotia Sea and east East Scotia Sea were yet to form. The reconstruction shows that the Jurassic igneous crust and continent–ocean transition of the Falkland Plateau Basin were contiguous with those of the central Scotia Sea and South Georgia^23^. The reconstructions in Fig. 8 present this as a consequence of their formation along the Skytrain-West Gondwana plate boundary in late Jurassic and early Cretaceous times. The reconstructed Falkland Plateau and central Scotia basins constitute a 600 km-wide barrier of thick oceanic lithosphere across South Georgia’s supposed path away from Tierra del Fuego in the Mesozoic back-arc interpretation of their origin. To sustain that interpretation, the South Georgia block must be envisaged to have ploughed through the barrier, requiring an implausible pair of closely-spaced subduction–transform edge propagation faults both north and south of a short (200 km-long) subduction zone at its leading eastern edge. Adding to this implausibility, as shown above, these two propagation faults and all their products must be hidden beneath the accretionary complex of the North Scotia Ridge.

To avoid this implausibility, the distribution of U–Pb dates on detrital zircon grains from South Georgia^9^ would need to be interpretable in terms of the extended continental margin scenario. For this, we note (Fig. 9b) that both the mid-Jurassic and late Albian peaks are relatable to in situ volcanic and plutonic rocks in South Georgia that can be understood in terms of breakup-related volcanism at the extended margin and to post-breakup explosive volcanic activity that was also responsible for the Annenkov Island formation^98^; there is no obligation for a location close to the sources of the zircons that delivered similar-aged peaks in Tierra del Fuego^96^. More prescriptively, the prominent Permian peak in the samples from South Georgia, which lacks any Permian outcrop of its own, fails to correlate closely with its 20 Myr-older counterpart in Tierra del Fuego. Instead, its age structure is almost identical to that of the population of U–Pb zircon ages from widespread ash layers of the Ecca group in the Karoo basin in South Africa^97^, which lies in the immediate hinterland of South Georgia in the extended margin interpretation (e.g. Figure 8c).

## Summary

A new magnetic anomaly data set from the Falkland Plateau Basin proves long-distance geological correlations to South Georgia, the Falkland Islands and Ellsworth Mountains to have been of little value in leading the construction of plate kinematic models of continental breakup in southwest Gondwana and the growth of the Scotia Sea. Instead of previous correlation-based schemes involving large rotations and long-distance translations of small continental blocks, the new data reveal the action of a previously-unrecognized plate, the Skytrain plate, starting at the active margin of southwest Gondwana in Jurassic times. The Skytrain plate and its motions provide a transformative and unifying context for a wide range of previously-disparate geological and geophysical observations in the Falkland Islands, Scotia and Weddell seas, Weddell Sea Embayment and Ellsworth Land regions.

## Supplementary information

Supplementary Information 1.

## Data Availability

Upon publication, the AIRLAFONIA magnetic anomaly data will be made publicly available via PANGAEA.
