# Supplementary material for: The Skytrain plate and tectonic evolution of southwest Gondwana since Jurassic times
Source: Sci Rep. 2020 Nov 17;10:19994. doi: 10.1038/s41598-020-77070-6 (PMC7672057; doi:10.1038/s41598-020-77070-6)
Supplement: Supplementary file 1 — Supplementary Information 1. [file 41598_2020_77070_MOESM1_ESM.pdf]

Supplementary materials to

The Skytrain plate and tectonic evolution of southwest Gondwana since Jurassic times

Graeme Eagles and Hannes Eisermann

Alfred Wegener Institut, Helmholtz Zentrum für Polar und Meeresforschung
Am Alten Hafen 26
27568 Bremerhaven
Germany

Contents:

1. Southwest Gondwana region plate topologies and rotations for 180 Ma reconstuction
2. Southwest Gondwana region plate topologies and rotations for 156 Ma reconstuction
3. Southwest Gondwana region plate topologies and rotations for 126 Ma reconstuction

1. Southwest Gondwana region plate topologies and rotations for 180 Ma reconstruction

>Part of East Antarctica on East Gondwana plate; rotation with respect to East Gondwana
lon: 0.0 lat: 0.0 ang: 0.0
-92.980 -72.769
-94.084 -72.318
-97.302 -71.468
-102.027 -71.295
-117.453 -73.303
-126.960 -73.042
-137.815 -74.403
-149.175 -75.418
-154.047 -76.513
-176.559 -73.877
175.832 -73.456
169.922 -70.446
146.361 -67.680
146.538 -69.838
150.192 -71.849
153.638 -74.018
157.989 -75.349
158.774 -77.380
155.630 -80.468
154.345 -82.787
157.351 -83.957
148.168 -87.319
99.750 -89.233
18.260 -87.255
-15.659 -86.646
-44.127 -84.924
-55.947 -83.217
-67.182 -83.682
-76.197 -83.524
-87.690 -83.599
-88.148 -82.020
-86.330 -81.235
-93.754 -80.562
-97.814 -79.514
-98.968 -77.735
-96.992 -77.519
-96.659 -77.144
-92.691 -73.241
-92.890 -72.950
>Part of East Antarctica on East Gondwana plate; rotation with respect to East Gondwana
lon: 0.0 lat: 0.0 ang: 0.0
17.229 -68.760
15.998 -68.412

14.798	-68.966
13.296	-69.162
11.465	-69.122
10.414	-69.072
8.484	-69.126
6.586	-69.276
5.391	-69.260
3.094	-69.200
0.313	-69.229
-0.618	-69.319
-1.654	-69.551
-2.701	-69.687
-4.287	-70.226
-6.767	-70.690
-8.892	-71.111
-11.821	-71.552
-14.982	-71.937
-18.319	-72.315
-19.294	-72.808
-21.050	-73.344
-21.854	-73.603
-25.356	-73.787
-27.318	-74.213
-27.988	-74.621
-29.903	-74.918
-31.552	-75.114
-34.822	-75.566
-37.347	-76.258
-38.433	-76.778
-39.088	-77.344
-39.783	-77.912
-40.124	-78.443
-39.967	-79.302
-40.153	-79.890
-42.005	-80.569
-44.439	-80.984
-46.591	-81.404
-48.782	-82.582
-51.833	-82.981
-55.787	-83.244
-48.128	-84.563
-36.000	-85.641
-9.601	-86.879
19.361	-87.289
98.199	-89.232
157.367	-83.943
154.774	-82.877

155.844	-80.504
158.837	-77.394
157.976	-75.521
153.436	-74.023
149.962	-71.792
146.550	-69.825
146.369	-67.737
139.420	-65.793
135.240	-65.410
117.965	-66.186
113.915	-65.550
109.512	-65.791
105.063	-64.962
101.503	-64.658
99.289	-64.714
93.751	-65.166
85.789	-65.601
82.778	-65.293
79.610	-65.389
77.995	-65.934
77.384	-66.595
75.861	-66.347
74.144	-66.715
73.117	-67.083
69.091	-67.270
59.113	-66.515
57.760	-66.004
56.512	-65.694
55.812	-65.500
55.080	-65.532
54.095	-65.138
52.165	-64.793
50.204	-65.424
47.900	-66.433
43.908	-66.917
41.155	-67.378
39.304	-67.697
35.927	-67.650
34.717	-66.610
35.088	-65.149
34.078	-64.753
33.269	-64.863
32.711	-65.847
32.688	-66.110
32.583	-66.414
31.960	-67.182
30.536	-67.866

29.499 -68.438
19.806 -69.590
18.342 -69.127
>Forrestal and Dufek magnetic anomalies; rotation with respect to East Gondwana lon: 0.0 lat: 0.0 ang: 0.0
-49.563 -83.445
-49.850 -83.282
-49.714 -83.170
-49.287 -83.028
-49.189 -82.955
-48.746 -82.939
-48.318 -82.949
-48.062 -82.921
-47.988 -82.875
-48.599 -82.811
-49.007 -82.781
-49.227 -82.749
-47.937 -82.622
-47.768 -82.451
-47.117 -82.385
-46.728 -82.323
-46.212 -82.206
-46.199 -82.126
-46.092 -82.053
-46.667 -81.935
-46.606 -81.869
-46.253 -81.861
-46.126 -81.791
-46.329 -81.754
-46.879 -81.732
-47.314 -81.720
-47.664 -81.804
-48.138 -81.827
-48.541 -81.840
-48.699 -81.879
-48.897 -81.947
-49.169 -81.967
-49.464 -82.028
-49.610 -82.112
-49.716 -82.168
-50.014 -82.251
-50.281 -82.281
-50.704 -82.316
-50.859 -82.387
-50.607 -82.420
-50.224 -82.452
-49.684 -82.398

-49.589 -82.326
-49.111 -82.271
-48.679 -82.195
-48.680 -82.145
-48.649 -82.079
-48.212 -82.013
-47.667 -81.957
-47.307 -81.942
-47.379 -82.021
-47.651 -82.119
-47.655 -82.133
-47.823 -82.261
-48.020 -82.323
-48.567 -82.375
-49.112 -82.457
-49.219 -82.556
-48.862 -82.659
-49.468 -82.713
-49.930 -82.775
-49.646 -82.868
-50.130 -82.869
-50.689 -82.963
-51.077 -83.158
-51.393 -83.284
-51.568 -83.463
-51.162 -83.498
-49.761 -83.476
>Pagano magnetic anomaly; rotation with respect to East Gondwana lon: 0.0 lat: 0.0 ang: 0.0
-90.946 -83.729
-88.537 -83.728
-87.187 -83.666
-86.009 -83.640
-84.505 -83.607
-82.663 -83.584
-81.358 -83.544
-79.801 -83.458
-78.508 -83.430
-77.651 -83.351
-78.135 -83.267
-80.189 -83.313
-81.626 -83.340
-82.771 -83.339
-84.222 -83.359
-85.831 -83.394
-86.819 -83.382
-87.777 -83.444

-89.751 -83.486
-90.745 -83.486
-91.582 -83.521
-91.601 -83.596
-91.272 -83.635
>Skytrain Plate; rotation with respect to East Gondwana lon: 127.9 lat: 78.4 ang: 53.2
-61.888 -69.607
-63.610 -69.196
-65.792 -68.983
-68.745 -69.266
-68.287 -69.674
-68.197 -70.107
-67.723 -70.730
-67.474 -71.361
-67.168 -72.203
-67.861 -72.810
-69.705 -73.142
-72.498 -73.140
-75.274 -72.839
-77.577 -72.492
-80.886 -72.160
-83.243 -72.125
-85.383 -72.151
-87.461 -72.246
-89.715 -72.382
-91.038 -72.718
-93.371 -73.518
-95.036 -75.341
-97.014 -77.558
-98.551 -77.670
-99.033 -78.567
-97.497 -79.589
-93.563 -80.597
-86.316 -81.268
-88.140 -82.054
-87.102 -83.562
-76.954 -83.504
-66.740 -83.625
-51.833 -82.981
-46.880 -81.874
-44.901 -80.703
-42.877 -79.818
-43.484 -78.989
-43.781 -78.381
-43.460 -78.206
-43.163 -78.058
-43.395 -77.899

-43.922 -77.423
-44.603 -76.932
-45.599 -76.324
-46.804 -75.276
-48.435 -74.723
-49.594 -74.190
-51.720 -73.753
-53.341 -73.250
-54.238 -72.892
-53.479 -72.105
-55.700 -71.693
-56.501 -71.368
-57.252 -71.105
-58.597 -70.791
-60.553 -70.021
>part of South America on West Gondwana plate; rotation with respect to East Gondwana
lon: 97.0 lat: 59.4 ang: 59.9
-48.430 -50.141
-47.935 -50.436
-47.652 -50.661
-47.263 -50.975
-46.661 -51.220
-45.918 -51.287
-45.352 -51.265
-44.715 -51.287
-44.397 -51.420
-44.008 -51.618
-43.512 -52.013
-42.982 -52.642
-41.920 -51.706
-41.496 -51.398
-41.460 -50.997
-41.248 -50.774
-40.823 -50.571
-40.753 -50.255
-40.788 -50.005
-40.540 -49.845
-40.010 -49.661
-40.470 -49.339
-41.718 -49.432
-44.891 -49.655
-48.321 -49.655
-51.580 -49.376
-54.066 -48.869
-55.953 -48.243
-58.440 -47.377
-59.126 -46.791

-59.555	-45.840
-59.555	-44.995
-59.297	-43.765
-58.855	-42.636
-58.130	-41.062
-58.638	-40.455
-60.742	-39.619
-61.758	-38.144
-64.589	-35.400
-65.460	-33.901
-65.822	-32.251
-66.476	-30.004
-71.120	-29.116
-71.628	-31.695
-71.846	-33.780
-72.589	-35.634
-74.390	-39.655
-75.289	-42.284
-75.338	-43.454
-75.587	-44.638
-75.736	-45.413
-75.636	-46.282
-75.686	-47.136
-75.786	-47.810
-75.984	-48.277
-75.935	-49.684
-75.786	-50.642
-75.487	-51.332
-75.487	-51.858
-75.139	-52.470
-74.080	-53.647
-72.203	-54.877
-70.103	-55.642
-68.315	-56.120
-68.012	-56.151
-67.450	-56.039
-66.947	-55.802
-66.528	-55.541
-65.992	-55.082
-65.242	-54.672
-64.879	-54.292
-62.961	-54.283
-61.420	-53.873
-59.730	-53.697
-58.195	-53.641
-56.851	-52.534
-56.320	-52.100

-55.259 -51.398
-53.737 -50.323
-52.923 -49.982
-51.791 -49.868
-51.154 -49.868
-50.411 -49.959
-49.633 -50.005
-48.925 -50.096
>East Falkland sheared margin magnetic anomaly; rotation with respect to East Gondwana
lon: 97.0 lat: 59.4 ang: 59.9
-55.750 -51.155
-56.023 -51.395
-56.183 -51.533
-56.433 -51.671
-56.638 -51.786
-56.865 -51.935
-57.047 -52.107
-57.229 -52.245
-57.411 -52.440
-57.502 -52.531
-57.866 -52.784
-57.935 -53.013
-58.299 -53.151
-58.663 -53.277
-58.594 -53.346
-58.208 -53.174
-57.775 -53.071
-57.570 -52.864
-57.479 -52.749
-57.411 -52.612
-57.229 -52.451
-57.115 -52.279
-56.956 -52.107
-56.729 -52.004
-56.615 -51.923
-56.501 -51.820
-56.319 -51.728
-56.183 -51.648
-56.001 -51.499
-56.069 -51.613
-56.137 -51.705
-56.319 -51.889
-56.569 -52.004
-56.706 -52.107
-56.820 -52.187
-56.933 -52.256
-57.047 -52.325

-57.115 -52.451
-56.842 -52.268
-56.592 -52.130
-56.456 -52.049
-56.137 -51.912
-56.001 -51.740
-55.887 -51.671
-55.864 -51.751
-55.682 -51.533
-55.568 -51.453
-55.546 -51.304
-55.546 -51.120
-55.591 -51.028
-55.659 -51.074
>part of Africa on West Gondwana plate; rotation with respect to East Gondwana lon: 145.8 lat: 6.6 ang: 56.3
12.927 -23.512
13.496 -25.386
13.609 -26.721
13.723 -27.788
13.950 -28.343
14.164 -29.551
15.203 -30.792
16.112 -31.629
16.826 -32.898
17.086 -33.553
18.124 -34.687
18.644 -35.275
19.033 -35.753
19.553 -36.070
19.942 -36.438
20.072 -37.117
22.020 -36.595
23.059 -36.438
23.384 -36.122
22.994 -35.805
23.254 -35.488
25.591 -35.008
26.111 -34.848
26.630 -34.580
27.170 -34.304
27.535 -33.953
28.546 -33.390
29.080 -32.988
29.557 -32.585
29.866 -31.988
30.512 -31.556

31.467 -30.541
32.113 -29.539
32.149 -29.153
32.603 -28.295
32.798 -26.848
32.668 -25.147
32.344 -24.138
32.084 -23.061
32.214 -21.854
34.032 -20.269
35.395 -19.717
36.499 -18.792
37.668 -17.675
38.772 -17.301
40.135 -17.177
40.914 -16.050
41.109 -15.106
40.914 -14.158
40.654 -13.269
40.654 -12.249
40.460 -11.161
13.709 -11.354
12.151 -12.887
11.177 -15.484
11.242 -17.800
11.956 -19.348
12.606 -21.125
12.800 -23.121
>Graham Land; rotation with respect to East Gondwana lon: 77.6 lat: -23.0 ang: 7.1
-70.559 -68.488
-70.480 -67.765
-69.779 -67.193
-68.446 -66.563
-67.545 -66.032
-67.038 -65.608
-66.012 -65.034
-64.063 -64.107
-61.430 -63.107
-60.320 -62.572
-59.062 -62.180
-57.380 -61.793
-55.944 -61.712
-54.594 -61.640
-53.654 -61.586
-52.921 -61.543
-51.886 -61.327
-50.964 -61.252

-49.292 -61.262
-48.236 -61.574
-46.788 -62.255
-46.888 -62.719
-48.440 -63.185
-49.572 -63.336
-50.449 -63.208
-51.454 -63.067
-53.045 -63.069
-54.274 -63.205
-55.500 -63.806
-55.741 -64.199
-56.905 -64.641
-58.078 -65.012
-58.675 -65.784
-58.371 -66.826
-57.488 -68.060
-59.521 -68.721
-60.525 -68.931
-61.240 -69.130
-62.122 -69.382
-63.783 -69.431
-66.043 -69.179
-67.963 -69.148
-69.213 -68.953
-70.058 -68.470
>Alexander Island region; rotation with respect to East Gondwana lon: 124.7 lat: 78.7 ang: 52.9
-70.156 -68.549
-69.477 -69.294
-69.190 -70.036
-68.590 -70.561
-68.409 -71.173
-68.753 -71.822
-69.464 -72.360
-71.081 -72.595
-72.951 -72.420
-75.259 -72.121
-76.860 -72.010
-78.549 -71.720
-80.914 -71.568
-83.028 -71.463
-85.635 -71.376
-83.741 -70.951
-80.588 -70.637
-74.284 -69.232
-72.346 -68.907

-71.171 -68.590
>Charcot magnetic anomaly; rotation with respect to East Gondwana lon: 124.7 lat: 78.7 ang: 52.9
-72.102 -69.548
-72.561 -69.383
-73.066 -69.330
-73.290 -69.516
-73.857 -69.574
-74.293 -69.510
-74.617 -69.488
-75.072 -69.671
-77.413 -70.102
-78.097 -70.126
-79.057 -70.350
-79.822 -70.529
-79.887 -70.492
-80.985 -70.569
-82.044 -70.857
-82.473 -70.980
-83.361 -70.964
-84.310 -70.997
-84.940 -71.163
-86.269 -71.445
-86.838 -71.633
-87.205 -71.925
-88.132 -71.885
-89.142 -71.736
-91.045 -71.657
-91.730 -71.912
-92.402 -71.931
-92.427 -72.113
-91.332 -72.202
-90.907 -72.204
-90.056 -72.388
-88.696 -72.423
-87.981 -72.482
-86.528 -72.383
-86.310 -72.196
-86.280 -71.935
-85.560 -71.789
-85.075 -72.037
-84.650 -71.764
-84.122 -71.591
-83.253 -71.556
-82.097 -71.309
-81.740 -71.183
-80.971 -71.093

-80.259	-71.212
-79.208	-71.156
-78.989	-70.873
-78.695	-70.695
-78.206	-70.583
-77.841	-70.531
-77.412	-70.340
-76.853	-70.350
-76.125	-70.371
-75.674	-70.547
-74.852	-70.521
-73.275	-70.215
-72.484	-70.080
-72.383	-69.862

>South Georgia microcontinent; rotation with respect to East Gondwana lon: 105.5 lat: 59.9 ang: 61.0

-37.926	-53.685
-37.608	-53.717
-37.316	-53.748
-36.864	-53.748
-36.492	-53.827
-35.855	-53.921
-35.589	-54.078
-35.403	-54.436
-35.350	-54.683
-35.111	-54.837
-34.713	-54.852
-34.315	-54.867
-34.155	-55.097
-34.474	-55.294
-34.899	-55.461
-35.138	-55.567
-35.563	-55.672
-35.828	-55.657
-36.120	-55.461
-36.545	-55.173
-37.077	-55.066
-37.448	-55.082
-37.687	-55.051
-38.165	-54.975
-38.537	-54.867
-38.989	-54.698
-39.387	-54.560
-39.440	-54.218
-39.334	-54.000
-39.281	-53.843
-39.307	-53.732

-38.936	-53.701
-38.378	-53.732
-37.926	-53.685

>

2. Southwest Gondwana region plate topologies and rotations for 156 Ma reconstruction

>Part of East Antarctica on East Gondwana plate; rotation with respect to East Gondwana
lon: 0.0 lat: 0.0 ang: 0.0

17.207	-68.748
15.977	-68.399
14.778	-68.954
13.277	-69.150
11.446	-69.109
10.396	-69.059
8.468	-69.113
6.570	-69.262
5.376	-69.246
3.081	-69.187
0.302	-69.216
-0.628	-69.305
-1.664	-69.537
-2.711	-69.673
-4.296	-70.212
-6.774	-70.675
-8.897	-71.096
-11.824	-71.538
-14.983	-71.922
-18.552	-72.208
-21.879	-72.627
-24.803	-73.127
-27.190	-73.453
-29.963	-74.011
-32.832	-74.653
-35.688	-75.266
-37.877	-75.750
-39.275	-76.128
-40.599	-76.686
-41.769	-77.170
-43.003	-77.818
-43.721	-78.628
-42.930	-79.431
-43.543	-80.030
-45.151	-80.735
-48.674	-81.437
-55.858	-81.854
-58.832	-82.094
-55.384	-83.396
-48.046	-84.550
-35.934	-85.627
-9.632	-86.865
19.180	-87.277

97.182	-89.238
157.351	-83.957
154.755	-82.892
155.831	-80.519
158.832	-77.408
157.970	-75.536
153.426	-74.037
149.951	-71.806
146.538	-69.838
146.357	-67.750
139.405	-65.806
135.224	-65.422
117.940	-66.196
113.889	-65.559
109.484	-65.800
105.035	-64.970
101.474	-64.664
99.259	-64.720
93.720	-65.171
85.756	-65.604
82.745	-65.295
79.576	-65.391
77.961	-65.935
77.348	-66.596
75.826	-66.348
74.108	-66.715
73.081	-67.083
69.054	-67.269
59.079	-66.511
57.727	-66.000
56.479	-65.690
55.780	-65.495
55.047	-65.527
54.063	-65.133
52.134	-64.788
50.172	-65.419
47.868	-66.426
43.876	-66.909
41.123	-67.370
39.273	-67.689
35.897	-67.641
34.689	-66.601
35.061	-65.140
34.053	-64.744
33.243	-64.854
32.685	-65.837
32.662	-66.101

32.556	-66.405
31.932	-67.173
30.508	-67.856
29.471	-68.428
19.782	-69.578
18.319	-69.115

>Part of East Antarctica on East Gondwana plate; rotation with respect to East Gondwana
lon: 0.0 lat: 0.0 ang: 0.0

17.229	-68.760
15.998	-68.412
14.798	-68.966
13.296	-69.162
11.465	-69.122
10.414	-69.072
8.484	-69.126
6.586	-69.276
5.391	-69.260
3.094	-69.200
0.313	-69.229
-0.618	-69.319
-1.654	-69.551
-2.701	-69.687
-4.287	-70.226
-6.767	-70.690
-8.892	-71.111
-11.821	-71.552
-14.982	-71.937
-18.319	-72.315
-19.294	-72.808
-21.050	-73.344
-21.854	-73.603
-25.356	-73.787
-27.318	-74.213
-27.988	-74.621
-29.903	-74.918
-31.552	-75.114
-34.822	-75.566
-37.347	-76.258
-38.433	-76.778
-39.088	-77.344
-39.783	-77.912
-40.124	-78.443
-39.967	-79.302
-40.153	-79.890
-42.005	-80.569
-44.439	-80.984
-46.591	-81.404

-48.782	-82.582
-51.833	-82.981
-55.787	-83.244
-48.128	-84.563
-36.000	-85.641
-9.601	-86.879
19.361	-87.289
98.199	-89.232
157.367	-83.943
154.774	-82.877
155.844	-80.504
158.837	-77.394
157.976	-75.521
153.436	-74.023
149.962	-71.792
146.550	-69.825
146.369	-67.737
139.420	-65.793
135.240	-65.410
117.965	-66.186
113.915	-65.550
109.512	-65.791
105.063	-64.962
101.503	-64.658
99.289	-64.714
93.751	-65.166
85.789	-65.601
82.778	-65.293
79.610	-65.389
77.995	-65.934
77.384	-66.595
75.861	-66.347
74.144	-66.715
73.117	-67.083
69.091	-67.270
59.113	-66.515
57.760	-66.004
56.512	-65.694
55.812	-65.500
55.080	-65.532
54.095	-65.138
52.165	-64.793
50.204	-65.424
47.900	-66.433
43.908	-66.917
41.155	-67.378
39.304	-67.697

35.927	-67.650
34.717	-66.610
35.088	-65.149
34.078	-64.753
33.269	-64.863
32.711	-65.847
32.688	-66.110
32.583	-66.414
31.960	-67.182
30.536	-67.866
29.499	-68.438
19.806	-69.590
18.342	-69.127

>Skytrain plate; rotation with respect to East Gondwana lon: 137.3 lat: 79.8 ang: 45.8

-63.915	-69.406
-62.190	-69.387
-59.436	-68.763
-57.496	-69.821
-55.740	-69.339
-54.550	-69.878
-53.730	-70.049
-51.790	-70.638
-50.596	-71.073
-53.425	-71.332
-53.079	-71.859
-51.878	-72.605
-49.017	-72.963
-46.755	-73.205
-44.914	-73.652
-46.016	-74.182
-44.276	-75.110
-45.379	-75.855
-45.792	-76.412
-45.587	-77.317
-44.908	-78.144
-43.963	-78.332
-44.135	-78.754
-43.563	-79.204
-43.332	-79.831
-42.708	-80.708
-45.901	-81.470
-47.433	-82.069
-49.186	-82.611
-53.070	-83.093
-67.699	-83.683
-75.994	-83.498
-88.093	-83.580

-88.187	-82.117
-95.877	-80.979
-97.901	-79.895
-97.531	-78.366
-96.340	-76.947
-94.929	-75.782
-93.762	-74.315
-93.144	-73.731
-92.696	-73.361
-91.231	-72.765
-89.767	-72.382
-86.354	-72.204
-84.893	-72.146
-83.320	-72.128
-80.992	-72.157
-77.534	-72.513
-75.065	-72.855
-72.420	-73.133
-69.597	-73.127
-67.870	-72.811
-67.205	-72.198
-67.435	-71.535
-67.705	-70.744
-68.326	-69.634
-69.136	-68.944
-66.300	-69.121
-64.614	-69.417

>part of South America on West Gondwana plate; rotation with respect to East Gondwana
lon: 95.7 lat: 62.6 ang: 56.5

-49.346	-51.098
-47.046	-52.339
-46.442	-52.585
-45.418	-53.002
-42.974	-52.635
-41.912	-51.699
-41.488	-51.391
-41.452	-50.990
-41.240	-50.767
-40.816	-50.564
-40.745	-50.248
-40.780	-49.997
-40.532	-49.838
-40.002	-49.654
-40.462	-49.331
-41.710	-49.425
-44.883	-49.648
-48.313	-49.648

-51.572	-49.369
-54.059	-48.862
-55.945	-48.236
-58.432	-47.369
-59.118	-46.783
-59.547	-45.833
-59.547	-44.987
-59.290	-43.757
-58.848	-42.628
-58.122	-41.054
-58.630	-40.447
-60.734	-39.610
-61.751	-38.135
-64.581	-35.391
-65.452	-33.892
-65.815	-32.242
-66.468	-29.995
-71.113	-29.106
-71.621	-31.685
-71.838	-33.771
-72.581	-35.625
-74.382	-39.646
-75.281	-42.276
-75.330	-43.446
-75.579	-44.630
-75.728	-45.405
-75.629	-46.274
-75.678	-47.129
-75.778	-47.803
-75.977	-48.270
-75.927	-49.677
-75.778	-50.635
-75.480	-51.325
-75.480	-51.852
-75.132	-52.463
-74.072	-53.641
-72.195	-54.871
-70.095	-55.636
-68.308	-56.114
-68.005	-56.145
-67.442	-56.033
-66.939	-55.795
-66.520	-55.535
-65.984	-55.076
-65.234	-54.666
-64.871	-54.285
-62.953	-54.276

-61.412 -53.867
-59.722 -53.690
-58.187 -53.635
-56.843 -52.527
-56.312 -52.093
-54.146 -50.577
-52.954 -51.246
-52.249 -51.634
-51.813 -51.288
-51.024 -51.540
-50.420 -51.130
>part of Africa on West Gondwana plate; rotation with respect to East Gondwana lon: 146.6 lat: 4.4 ang: 52.1
12.927 -23.512
13.496 -25.386
13.609 -26.721
13.723 -27.788
13.950 -28.343
14.164 -29.551
15.203 -30.792
16.112 -31.629
16.826 -32.898
17.086 -33.553
18.124 -34.687
18.644 -35.275
19.033 -35.753
19.553 -36.070
19.942 -36.438
20.072 -37.117
22.020 -36.595
23.059 -36.438
23.384 -36.122
22.994 -35.805
23.254 -35.488
25.591 -35.008
26.111 -34.848
26.630 -34.580
27.170 -34.304
27.535 -33.953
28.546 -33.390
29.080 -32.988
29.557 -32.585
29.866 -31.988
30.512 -31.556
31.467 -30.541
32.113 -29.539
32.149 -29.153

32.603	-28.295
32.798	-26.848
32.668	-25.147
32.344	-24.138
32.084	-23.061
32.214	-21.854
34.032	-20.269
35.395	-19.717
36.499	-18.792
37.668	-17.675
38.772	-17.301
40.135	-17.177
40.914	-16.050
41.109	-15.106
40.914	-14.158
40.654	-13.269
40.654	-12.249
40.460	-11.161
13.709	-11.354
12.151	-12.887
11.177	-15.484
11.242	-17.800
11.956	-19.348
12.606	-21.125
12.800	-23.121

>Graham Land; rotation with respect to East Gondwana lon: 46.5 lat: -63.4 ang: 10.7

-70.559	-68.488
-70.480	-67.765
-69.779	-67.193
-68.446	-66.563
-67.545	-66.032
-67.038	-65.608
-66.012	-65.034
-64.063	-64.107
-61.430	-63.107
-60.320	-62.572
-59.062	-62.180
-57.380	-61.793
-55.944	-61.712
-54.594	-61.640
-53.654	-61.586
-52.921	-61.543
-51.886	-61.327
-50.964	-61.252
-49.292	-61.262
-48.236	-61.574
-46.788	-62.255

-46.888 -62.719
-48.440 -63.185
-49.572 -63.336
-50.449 -63.208
-51.454 -63.067
-53.045 -63.069
-54.274 -63.205
-55.500 -63.806
-55.741 -64.199
-56.905 -64.641
-58.078 -65.012
-58.675 -65.784
-58.371 -66.826
-57.488 -68.060
-59.521 -68.721
-60.525 -68.931
-61.240 -69.130
-62.122 -69.382
-63.783 -69.431
-66.043 -69.179
-67.963 -69.148
-69.213 -68.953
-70.058 -68.470
>Alexander Island region; rotation with respect to East Gondwana lon: 133.6 lat: 80.3 ang: 45.5
-70.156 -68.549
-69.477 -69.294
-69.190 -70.036
-68.590 -70.561
-68.409 -71.173
-68.753 -71.822
-69.464 -72.360
-71.081 -72.595
-72.951 -72.420
-75.259 -72.121
-76.860 -72.010
-78.549 -71.720
-80.914 -71.568
-83.028 -71.463
-85.635 -71.376
-83.741 -70.951
-80.588 -70.637
-74.284 -69.232
-72.346 -68.907
-71.171 -68.590
>South Georgia microcontinent; rotation with respect to East Gondwana lon: 106.1 lat: 60.9 ang: 55.4

-37.926	-53.685
-37.608	-53.717
-37.316	-53.748
-36.864	-53.748
-36.492	-53.827
-35.855	-53.921
-35.589	-54.078
-35.403	-54.436
-35.350	-54.683
-35.111	-54.837
-34.713	-54.852
-34.315	-54.867
-34.155	-55.097
-34.474	-55.294
-34.899	-55.461
-35.138	-55.567
-35.563	-55.672
-35.828	-55.657
-36.120	-55.461
-36.545	-55.173
-37.077	-55.066
-37.448	-55.082
-37.687	-55.051
-38.165	-54.975
-38.537	-54.867
-38.989	-54.698
-39.387	-54.560
-39.440	-54.218
-39.334	-54.000
-39.281	-53.843
-39.307	-53.732
-38.936	-53.701
-38.378	-53.732
-37.926	-53.685

>

3. Southwest Gondwana region plate topologies and rotations for 126 Ma reconstruction

>Part of East Antarctica plate; rotation with respect to East Antarctica lon: 0.0 lat: 0.0 ang: 0.0

20.659	-63.187
17.425	-64.920
14.969	-64.990
13.586	-64.745
10.592	-64.693
7.714	-65.116
5.109	-65.752
2.421	-66.113
-1.828	-66.222
-4.338	-66.460
-6.705	-66.637
-9.312	-67.307
-10.795	-67.235
-12.445	-67.059
-13.485	-67.455
-14.611	-67.571
-15.555	-67.684
-17.994	-67.889
-19.597	-68.128
-18.511	-69.800
-20.804	-70.138
-19.999	-70.581
-21.832	-71.133
-24.200	-71.606
-26.284	-72.290
-27.871	-72.855
-32.021	-74.067
-36.553	-75.054
-40.089	-75.791
-44.603	-76.358
-50.644	-76.692
-53.781	-76.440
-60.124	-74.562
-62.238	-73.385
-65.140	-73.606
-67.307	-73.964
-70.038	-74.197
-76.486	-73.941
-82.015	-73.383
-85.637	-73.048
-90.412	-72.706
-97.638	-71.432
-102.400	-71.276

-116.101	-73.218
-127.341	-73.082
-148.704	-75.332
-154.964	-76.455
-174.944	-74.085
174.935	-73.282
170.047	-70.581
160.281	-69.699
158.756	-77.425
157.903	-75.552
153.361	-74.052
149.891	-71.819
146.482	-69.851
146.306	-67.762
139.355	-65.815
135.173	-65.431
117.884	-66.197
115.557	-65.868
113.944	-65.574
111.308	-65.743
109.480	-65.790
105.103	-64.989
101.508	-64.658
99.213	-64.727
94.417	-65.124
85.582	-65.604
82.697	-65.328
79.748	-65.314
77.962	-65.902
77.249	-66.599
75.966	-66.387
74.237	-66.679
72.843	-67.066
69.141	-67.266
59.154	-66.507
57.798	-66.018
55.957	-65.578
54.995	-65.542
54.475	-65.282
53.875	-65.073
52.204	-64.784
50.157	-65.447
47.893	-66.399
43.833	-66.893
39.236	-67.676
35.768	-67.661
34.590	-66.626

35.013 -65.070
33.994 -64.568
28.780 -63.533
24.785 -62.987
23.508 -63.799
21.111 -63.279
>Eastern Palmer Land Shear Zone magnetic anomaly; rotation with respect to East Antarctica lon: 0.0 lat: 0.0 ang: 0.0
-66.614 -68.659
-65.384 -68.947
-64.389 -69.361
-63.600 -69.802
-63.134 -70.302
-62.946 -70.586
-62.730 -71.143
-62.578 -71.714
-63.274 -72.480
-64.195 -73.023
-64.722 -73.299
-65.500 -73.584
-66.983 -73.917
-68.713 -74.111
-70.574 -74.222
-73.494 -74.166
-76.006 -73.997
>Skytrain plate; rotation with respect to East Antarctica lon: 110.0 lat: -1.3 ang: 0.05
-68.800 -69.019
-67.976 -69.118
-66.205 -69.170
-64.756 -69.383
-63.475 -69.445
-61.906 -69.412
-58.390 -68.398
-57.599 -68.086
-58.436 -66.784
-58.686 -65.729
-57.976 -65.003
-55.731 -64.170
-55.663 -63.827
-54.091 -63.186
-52.969 -63.020
-51.884 -63.067
-51.110 -64.122
-50.578 -64.559
-50.106 -65.182
-49.535 -65.865
-49.154 -66.046

-48.232	-66.409
-46.779	-66.745
-45.653	-66.963
-45.226	-67.154
-44.348	-67.573
-43.569	-67.673
-42.387	-67.826
-41.712	-68.035
-40.676	-68.273
-39.825	-68.363
-38.934	-68.363
-38.416	-68.363
-37.503	-68.615
-36.509	-68.688
-34.766	-68.802
-32.987	-68.780
-30.810	-68.690
-29.093	-68.532
-28.001	-68.617
-27.406	-68.421
-26.898	-68.565
-25.175	-68.450
-24.626	-68.527
-24.070	-68.473
-23.462	-68.425
-22.872	-68.561
-21.220	-68.244
-20.865	-68.295
-20.360	-68.325
-19.444	-68.416
-18.471	-69.758
-20.828	-70.119
-19.969	-70.554
-21.866	-71.160
-24.285	-71.594
-26.262	-72.310
-27.946	-72.876
-31.679	-73.996
-36.646	-75.057
-37.386	-75.284
-40.067	-75.780
-43.034	-76.187
-45.353	-76.369
-50.722	-76.683
-53.871	-76.430
-57.140	-75.512
-59.811	-74.602

-60.863 -74.149
-62.303 -73.358
-65.385 -73.640
-70.211 -74.186
-76.406 -73.930
-81.256 -73.452
-86.069 -73.004
-90.831 -72.642
-89.498 -72.303
-87.372 -72.199
-85.544 -72.127
-83.422 -72.064
-81.026 -72.075
-77.887 -72.423
-75.367 -72.757
-72.670 -73.040
-69.766 -73.084
-67.893 -72.762
-67.196 -72.205
-67.712 -70.727
-68.158 -70.113
-68.335 -69.614
-69.042 -69.023
>part of South America plate; rotation rotation with respect to East Antarctica lon: 90.1 lat: 72.6 ang: 47.9
-48.250 -53.242
-47.173 -53.242
-45.225 -53.220
-44.333 -52.857
-42.988 -52.648
-42.036 -52.909
-40.222 -53.224
-37.976 -53.187
-36.223 -53.334
-35.177 -53.628
-35.177 -52.045
-35.731 -52.083
-35.761 -51.511
-35.638 -50.107
-35.269 -49.128
-41.724 -49.438
-44.897 -49.661
-48.327 -49.661
-51.586 -49.382
-54.073 -48.876
-54.832 -48.621
-54.525 -46.349

-54.925	-46.242
-54.709	-44.662
-54.525	-43.777
-53.940	-41.944
-53.940	-41.042
-53.510	-41.042
-51.418	-37.554
-50.464	-36.816
-49.173	-34.761
-48.988	-33.534
-54.463	-32.575
-61.323	-32.835
-63.968	-33.069
-64.614	-32.653
-65.829	-32.260
-66.482	-30.013
-71.127	-29.124
-71.635	-31.703
-71.852	-33.788
-72.595	-35.642
-74.396	-39.662
-75.295	-42.292
-75.345	-43.462
-75.593	-44.645
-75.742	-45.420
-75.643	-46.288
-75.692	-47.143
-75.792	-47.817
-75.991	-48.284
-75.941	-49.690
-75.792	-50.648
-75.494	-51.338
-75.494	-51.864
-75.146	-52.476
-74.086	-53.653
-72.209	-54.883
-70.109	-55.648
-68.322	-56.125
-68.019	-56.157
-67.456	-56.044
-66.953	-55.807
-66.534	-55.547
-65.875	-55.189
-65.321	-55.065
-63.691	-54.888
-63.168	-54.764
-62.430	-54.675

-61.230 -54.245
-59.662 -54.245
-58.185 -54.173
-56.463 -54.173
-54.863 -54.119
-52.956 -53.646
-51.726 -53.536
-50.649 -53.242
-48.834 -53.187
>Maurice Ewing Bank, rotation with respect to East Antarctica lon: 90.1 lat: 72.6 ang: 47.9
-47.506 -50.425
-47.210 -50.632
-46.558 -50.858
-46.024 -51.027
-45.312 -51.064
-44.986 -51.083
-44.423 -51.250
-43.801 -51.603
-43.504 -51.860
-43.208 -52.171
-42.555 -52.226
-41.963 -52.007
-41.607 -51.621
-41.221 -51.381
-40.777 -51.139
-40.451 -50.914
-40.184 -50.708
-40.006 -50.481
-39.858 -50.197
-39.858 -49.872
-39.947 -49.565
-40.095 -49.410
-42.882 -49.565
-45.579 -49.642
-46.587 -49.642
-47.239 -49.584
-49.819 -49.565
-50.501 -49.449
-51.627 -49.313
-52.516 -49.255
-52.961 -49.526
-52.754 -49.680
-51.508 -49.910
-50.560 -49.910
-49.493 -49.968
-48.603 -50.121
-48.040 -50.311

-47.714 -50.406
>East Falkland sheared margin magnetic anomaly, rotation with respect to East Antarctica
lon: 90.1 lat: 72.6 ang: 47.9
-55.750 -51.155
-56.023 -51.395
-56.183 -51.533
-56.433 -51.671
-56.638 -51.786
-56.865 -51.935
-57.047 -52.107
-57.229 -52.245
-57.411 -52.440
-57.502 -52.531
-57.866 -52.784
-57.935 -53.013
-58.299 -53.151
-58.663 -53.277
-58.594 -53.346
-58.208 -53.174
-57.775 -53.071
-57.570 -52.864
-57.479 -52.749
-57.411 -52.612
-57.229 -52.451
-57.115 -52.279
-56.956 -52.107
-56.729 -52.004
-56.615 -51.923
-56.501 -51.820
-56.319 -51.728
-56.183 -51.648
-56.001 -51.499
-56.069 -51.613
-56.137 -51.705
-56.319 -51.889
-56.569 -52.004
-56.706 -52.107
-56.820 -52.187
-56.933 -52.256
-57.047 -52.325
-57.115 -52.451
-56.842 -52.268
-56.592 -52.130
-56.456 -52.049
-56.137 -51.912
-56.001 -51.740
-55.887 -51.671

-55.864 -51.751
-55.682 -51.533
-55.568 -51.453
-55.546 -51.304
-55.546 -51.120
-55.591 -51.028
-55.659 -51.074
>Magnetic reversal anomaly area 1a, Falkland Plateau Basin, rotation with respect to East Antarctica lon: 90.1 lat: 72.6 ang: 47.9
-50.742 -52.471
-49.882 -52.685
>Magnetic reversal anomaly area 1a, Falkland Plateau Basin, rotation with respect to East Antarctica lon: 90.1 lat: 72.6 ang: 47.9
-50.570 -52.278
-50.076 -52.460
-49.624 -52.631
>Magnetic reversal anomaly area 1a, Falkland Plateau Basin, rotation with respect to East Antarctica lon: 90.1 lat: 72.6 ang: 47.9
-50.377 -52.139
-49.925 -52.364
-49.839 -52.406
>Magnetic reversal anomaly area 1a, Falkland Plateau Basin, rotation with respect to East Antarctica lon: 90.1 lat: 72.6 ang: 47.9
-50.678 -51.807
-50.205 -52.032
-49.947 -52.096
-49.624 -52.299
-49.194 -52.546
-49.087 -52.621
>Magnetic reversal anomaly area 1a, Falkland Plateau Basin, rotation with respect to East Antarctica lon: 90.1 lat: 72.6 ang: 47.9
-50.291 -51.464
-49.796 -51.689
-49.474 -51.839
-49.216 -51.871
-48.850 -52.032
-48.463 -52.203
-48.184 -52.353
-47.969 -52.503
>Magnetic reversal anomaly area 1a, Falkland Plateau Basin, rotation with respect to East Antarctica lon: 90.1 lat: 72.6 ang: 47.9
-49.624 -51.507
-49.237 -51.732
>Magnetic reversal anomaly area 1a, Falkland Plateau Basin, rotation with respect to East Antarctica lon: 90.1 lat: 72.6 ang: 47.9
-49.366 -51.442
-48.872 -51.742

-48.721 -51.796
-48.420 -51.957
-48.184 -52.085
-47.904 -52.235
-47.668 -52.374
-47.280 -52.460
>Magnetic reversal anomaly area 1a, Falkland Plateau Basin, rotation with respect to East Antarctica lon: 90.1 lat: 72.6 ang: 47.9
-48.248 -51.635
-47.840 -51.764
-47.603 -51.892
-47.237 -52.053
-47.001 -52.235
>Magnetic reversal anomaly area 1a, Falkland Plateau Basin, rotation with respect to East Antarctica lon: 90.1 lat: 72.6 ang: 47.9
-47.496 -50.511
-47.259 -50.789
-46.958 -50.993
-46.614 -51.228
>Magnetic reversal anomaly area 1a, Falkland Plateau Basin, rotation with respect to East Antarctica lon: 90.1 lat: 72.6 ang: 47.9
-47.904 -50.489
-47.582 -50.832
-47.345 -51.100
>Magnetic reversal anomaly area 1a, Falkland Plateau Basin, rotation with respect to East Antarctica lon: 90.1 lat: 72.6 ang: 47.9
-47.904 -50.757
-47.775 -51.132
>Fracture zone or isochron offset, Falkland Plateau Basin, rotation with respect to East Antarctica lon: 90.1 lat: 72.6 ang: 47.9
-52.679 -52.367
-52.202 -52.070
-51.103 -51.453
-50.455 -51.175
-49.578 -50.858
-48.840 -50.516
-48.408 -50.206
>Fracture zone or isochron offset, Falkland Plateau Basin, rotation with respect to East Antarctica lon: 90.1 lat: 72.6 ang: 47.9
-58.332 -53.285
-57.128 -52.353
-56.440 -51.817
-55.902 -51.528
-55.400 -51.137
-54.713 -50.731
>Fracture zone or isochron offset, Falkland Plateau Basin, rotation with respect to East Antarctica lon: 90.1 lat: 72.6 ang: 47.9

-52.536	-53.068
-51.558	-52.450
-50.700	-51.998

>part of Africa plate; rotation with respect to East Antarctica lon: 147.2 lat: 3.8 ang: 38.5

11.119	-23.379
11.819	-25.388
11.856	-26.256
12.280	-27.513
13.036	-32.109
13.221	-32.891
13.479	-33.773
13.645	-34.555
14.253	-35.540
14.880	-36.484
16.539	-37.915
17.609	-39.332
21.979	-36.929
23.048	-36.444
23.372	-36.128
24.210	-35.841
25.242	-35.314
25.580	-35.014
26.099	-34.854
26.619	-34.586
27.158	-34.310
27.524	-33.959
28.535	-33.396
29.068	-32.994
29.546	-32.591
29.855	-31.995
30.501	-31.563
31.456	-30.547
32.102	-29.545
32.137	-29.160
32.592	-28.301
32.787	-26.855
32.657	-25.153
32.332	-24.145
32.073	-23.068
32.202	-21.861
34.020	-20.276
35.384	-19.724
36.488	-18.799
37.656	-17.682
38.760	-17.309
40.124	-17.184
40.903	-16.057

41.097 -15.113
40.903 -14.165
40.643 -13.276
40.643 -12.256
40.448 -11.169
13.698 -11.361
12.140 -12.894
11.166 -15.491
11.231 -17.807
11.469 -19.251
11.506 -20.975
11.321 -22.559
11.119 -23.379
>Graham Land; rotation with respect to East Antarctica lon: 0.0 lat: 0.0 ang: 0.0
-70.559 -68.488
-70.480 -67.765
-69.779 -67.193
-68.446 -66.563
-67.545 -66.032
-67.038 -65.608
-66.012 -65.034
-64.063 -64.107
-61.430 -63.107
-60.320 -62.572
-59.062 -62.180
-57.380 -61.793
-55.944 -61.712
-54.594 -61.640
-53.654 -61.586
-52.921 -61.543
-51.886 -61.327
-50.964 -61.252
-49.292 -61.262
-48.236 -61.574
-46.788 -62.255
-46.888 -62.719
-48.440 -63.185
-49.572 -63.336
-50.449 -63.208
-51.454 -63.067
-53.045 -63.069
-54.274 -63.205
-55.500 -63.806
-55.741 -64.199
-56.905 -64.641
-58.078 -65.012
-58.675 -65.784

-58.371 -66.826
-57.488 -68.060
-59.521 -68.721
-60.525 -68.931
-61.240 -69.130
-62.122 -69.382
-63.783 -69.431
-66.043 -69.179
-67.963 -69.148
-69.213 -68.953
-70.058 -68.470
>Alexander Island region; rotation with respect to East Gondwana lon: -15.0 lat: -18.9 ang: 0.7
-70.156 -68.549
-69.477 -69.294
-69.190 -70.036
-68.590 -70.561
-68.409 -71.173
-68.753 -71.822
-69.464 -72.360
-71.081 -72.595
-72.951 -72.420
-75.259 -72.121
-76.860 -72.010
-78.549 -71.720
-80.914 -71.568
-83.028 -71.463
-85.635 -71.376
-83.741 -70.951
-80.588 -70.637
-74.284 -69.232
-72.346 -68.907
-71.171 -68.590
>South Georgia microcontinent; rotation with respect to East Gondwana lon: 107.7 lat: 70.6 ang: 46.2
-37.926 -53.685
-37.608 -53.717
-37.316 -53.748
-36.864 -53.748
-36.492 -53.827
-35.855 -53.921
-35.589 -54.078
-35.403 -54.436
-35.350 -54.683
-35.111 -54.837
-34.713 -54.852
-34.315 -54.867

-34.155 -55.097
-34.474 -55.294
-34.899 -55.461
-35.138 -55.567
-35.563 -55.672
-35.828 -55.657
-36.120 -55.461
-36.545 -55.173
-37.077 -55.066
-37.448 -55.082
-37.687 -55.051
-38.165 -54.975
-38.537 -54.867
-38.989 -54.698
-39.387 -54.560
-39.440 -54.218
-39.334 -54.000
-39.281 -53.843
-39.307 -53.732
-38.936 -53.701
-38.378 -53.732
-37.926 -53.685
>Magnetic reversal anomaly edge in central Scotia Sea pre-Drake Passage location; rotation with respect to East Antarctica lon: 90.1 lat: 72.6 ang: 47.9
-51.154 -56.887
-51.279 -56.861
-51.419 -56.823
-51.569 -56.794
-51.640 -56.767
-51.773 -56.730
-51.869 -56.700
-51.950 -56.683
-52.058 -56.648
-52.131 -56.609
-52.299 -56.556
>Magnetic reversal anomaly edge in central Scotia Sea pre-Drake Passage location; rotation with respect to East Antarctica lon: 90.1 lat: 72.6 ang: 47.9
-51.042 -57.196
-51.203 -57.178
-51.260 -57.168
-51.382 -57.155
-51.470 -57.156
-51.485 -57.143
>Magnetic reversal anomaly edge in central Scotia Sea pre-Drake Passage location; rotation with respect to East Antarctica lon: 90.1 lat: 72.6 ang: 47.9
-51.583 -57.189
-51.770 -57.168

-51.962 -57.143
>Magnetic reversal anomaly edge in central Scotia Sea pre-Drake Passage location; rotation with respect to East Antarctica lon: 90.1 lat: 72.6 ang: 47.9
-52.182 -57.141
-52.563 -57.087
>Magnetic reversal anomaly edge in central Scotia Sea pre-Drake Passage location; rotation with respect to East Antarctica lon: 90.1 lat: 72.6 ang: 47.9
-50.785 -57.060
-51.417 -57.060
-51.497 -56.985
-51.893 -56.976
-52.009 -56.976
-52.138 -56.848
-52.380 -56.839
-52.751 -56.867
>Magnetic reversal anomaly edge in central Scotia Sea pre-Drake Passage location; rotation with respect to East Antarctica lon: 90.1 lat: 72.6 ang: 47.9
-50.772 -56.983
-51.088 -56.983
-51.109 -56.937
-51.278 -56.935
-51.432 -56.936
-51.473 -56.878
-51.591 -56.872
-51.702 -56.875
-51.790 -56.865
-51.919 -56.859
>Magnetic reversal anomaly edge in central Scotia Sea pre-Drake Passage location; rotation with respect to East Antarctica lon: 90.1 lat: 72.6 ang: 47.9
-51.990 -56.790
-52.430 -56.657
>Magnetic reversal anomaly edge in central Scotia Sea pre-Drake Passage location; rotation with respect to East Antarctica lon: 90.1 lat: 72.6 ang: 47.9
-50.766 -56.911
-51.079 -56.884
>Magnetic reversal anomaly edge in central Scotia Sea pre-Drake Passage location; rotation with respect to East Antarctica lon: 90.1 lat: 72.6 ang: 47.9
-50.884 -56.695
-51.087 -56.702
-51.257 -56.714
-51.406 -56.697
-51.453 -56.636
-51.587 -56.624
-51.757 -56.613
>Magnetic reversal anomaly edge in central Scotia Sea pre-Drake Passage location; rotation with respect to East Antarctica lon: 90.1 lat: 72.6 ang: 47.9
-54.199 -56.421

-54.018 -56.419
-53.847 -56.408
-53.681 -56.374
-53.511 -56.401
-53.393 -56.385
-53.248 -56.389
-53.107 -56.389
-52.997 -56.398
-52.839 -56.404
>Magnetic reversal anomaly edge in central Scotia Sea pre-Drake Passage location; rotation with respect to East Antarctica lon: 90.1 lat: 72.6 ang: 47.9
-52.670 -56.453
-52.374 -56.451
>Magnetic reversal anomaly edge in central Scotia Sea pre-Drake Passage location; rotation with respect to East Antarctica lon: 90.1 lat: 72.6 ang: 47.9
-53.889 -56.264
-53.689 -56.263
-53.538 -56.268
-53.401 -56.260
-53.250 -56.266
-53.110 -56.285
-53.015 -56.299
-52.903 -56.304
-52.801 -56.319
>Magnetic reversal anomaly edge in central Scotia Sea pre-Drake Passage location; rotation with respect to East Antarctica lon: 90.1 lat: 72.6 ang: 47.9
-52.185 -56.279
-52.333 -56.263
-52.417 -56.273
-52.481 -56.266
-52.567 -56.245
>Magnetic reversal anomaly edge in central Scotia Sea pre-Drake Passage location; rotation with respect to East Antarctica lon: 90.1 lat: 72.6 ang: 47.9
-52.745 -56.236
-52.903 -56.230
-53.061 -56.205
-53.141 -56.204
-53.207 -56.220
-53.348 -56.204
-53.464 -56.192
-53.555 -56.185
-53.629 -56.189
-53.719 -56.179
-53.832 -56.178
-53.974 -56.147
-54.125 -56.103
-54.226 -56.088

-54.285 -56.085
-54.408 -56.095
-54.562 -56.078
-54.708 -56.039
-54.800 -56.017
-54.849 -56.008
>Magnetic reversal anomaly edge in central Scotia Sea pre-Drake Passage location; rotation with respect to East Antarctica lon: 90.1 lat: 72.6 ang: 47.9
-53.792 -56.052
-53.605 -56.070
-53.531 -56.056
-53.406 -56.052
-53.284 -56.045
-53.137 -56.048
-52.999 -56.052
-52.921 -56.038
-52.841 -56.053
-52.800 -56.081
-52.680 -56.092
-52.539 -56.121
-52.449 -56.106
-52.280 -56.117
-52.152 -56.124
>Magnetic reversal anomaly edge in central Scotia Sea pre-Drake Passage location; rotation with respect to East Antarctica lon: 90.1 lat: 72.6 ang: 47.9
-53.589 -55.925
-53.439 -55.934
-53.420 -55.937
>Magnetic reversal anomaly edge in central Scotia Sea pre-Drake Passage location; rotation with respect to East Antarctica lon: 90.1 lat: 72.6 ang: 47.9
-53.327 -55.900
-53.187 -55.900
>Magnetic reversal anomaly edge in central Scotia Sea pre-Drake Passage location; rotation with respect to East Antarctica lon: 90.1 lat: 72.6 ang: 47.9
-53.049 -55.888
-52.880 -55.903
-52.738 -55.899
>Magnetic reversal anomaly edge in central Scotia Sea pre-Drake Passage location; rotation with respect to East Antarctica lon: 90.1 lat: 72.6 ang: 47.9
-52.477 -55.936
-52.348 -55.950
-52.191 -55.959
>Magnetic reversal anomaly edge in central Scotia Sea pre-Drake Passage location; rotation with respect to East Antarctica lon: 90.1 lat: 72.6 ang: 47.9
-53.616 -55.868
-53.450 -55.871

>Magnetic reversal anomaly edge in central Scotia Sea pre-Drake Passage location; rotation with respect to East Antarctica lon: 90.1 lat: 72.6 ang: 47.9
-53.294 -55.845
-53.187 -55.845
>Magnetic reversal anomaly edge in central Scotia Sea pre-Drake Passage location; rotation with respect to East Antarctica lon: 90.1 lat: 72.6 ang: 47.9
-53.069 -55.811
-52.904 -55.818
-52.797 -55.818
-52.726 -55.826
>Magnetic reversal anomaly edge in central Scotia Sea pre-Drake Passage location; rotation with respect to East Antarctica lon: 90.1 lat: 72.6 ang: 47.9
-52.438 -55.862
-52.158 -55.885
>Magnetic reversal anomaly edge in central Scotia Sea pre-Drake Passage location; rotation with respect to East Antarctica lon: 90.1 lat: 72.6 ang: 47.9
-52.923 -55.764
-53.099 -55.769
-53.206 -55.765
-53.320 -55.783
-53.439 -55.783
-53.560 -55.788
-53.658 -55.790
>Magnetic reversal anomaly edge in central Scotia Sea pre-Drake Passage location; rotation with respect to East Antarctica lon: 90.1 lat: 72.6 ang: 47.9
-53.706 -55.823
-53.928 -55.791
-54.139 -55.744
>Magnetic reversal anomaly edge in central Scotia Sea pre-Drake Passage location; rotation with respect to East Antarctica lon: 90.1 lat: 72.6 ang: 47.9
-54.166 -55.596
-54.017 -55.596
-53.808 -55.619
-53.614 -55.648
-53.349 -55.658
-53.226 -55.669
-53.102 -55.662
-52.953 -55.665
>Magnetic reversal anomaly edge in central Scotia Sea pre-Drake Passage location; rotation with respect to East Antarctica lon: 90.1 lat: 72.6 ang: 47.9
-52.667 -55.715
-52.425 -55.730
>Magnetic reversal anomaly edge in central Scotia Sea pre-Drake Passage location; rotation with respect to East Antarctica lon: 90.1 lat: 72.6 ang: 47.9
-53.848 -55.423
-53.657 -55.448
-53.609 -55.465

>Magnetic reversal anomaly edge in central Scotia Sea pre-Drake Passage location; rotation with respect to East Antarctica lon: 90.1 lat: 72.6 ang: 47.9
-53.542 -55.524
-53.315 -55.541
-53.184 -55.567
-53.095 -55.581
>Magnetic reversal anomaly edge in central Scotia Sea pre-Drake Passage location; rotation with respect to East Antarctica lon: 90.1 lat: 72.6 ang: 47.9
-52.803 -55.625
-52.616 -55.646
-52.477 -55.649
-52.322 -55.667
-52.204 -55.672
>Magnetic reversal anomaly edge in central Scotia Sea pre-Drake Passage location; rotation with respect to East Antarctica lon: 90.1 lat: 72.6 ang: 47.9
-51.794 -55.670
-51.606 -55.667
>Magnetic reversal anomaly edge in central Scotia Sea pre-Drake Passage location; rotation with respect to East Antarctica lon: 90.1 lat: 72.6 ang: 47.9
-53.765 -55.318
-53.642 -55.347
>Magnetic reversal anomaly edge in central Scotia Sea pre-Drake Passage location; rotation with respect to East Antarctica lon: 90.1 lat: 72.6 ang: 47.9
-53.554 -55.403
-53.355 -55.418
-53.201 -55.423
-53.187 -55.448
-53.068 -55.463
-52.987 -55.474
-52.686 -55.510
-52.596 -55.508
-52.448 -55.518
-52.253 -55.525
-52.097 -55.538
-51.934 -55.570
-51.843 -55.532
-51.795 -55.526
>Magnetic reversal anomaly edge in central Scotia Sea pre-Drake Passage location; rotation with respect to East Antarctica lon: 90.1 lat: 72.6 ang: 47.9
-51.801 -55.515
-51.551 -55.531
>Magnetic reversal anomaly edge in central Scotia Sea pre-Drake Passage location; rotation with respect to East Antarctica lon: 90.1 lat: 72.6 ang: 47.9
-53.318 -55.337
-53.218 -55.361
-53.099 -55.360
-52.940 -55.372

-52.782 -55.391
-52.638 -55.399
-52.499 -55.402
-52.309 -55.415
-52.136 -55.419
-52.029 -55.419
-51.916 -55.423
>Magnetic reversal anomaly edge in central Scotia Sea pre-Drake Passage location; rotation with respect to East Antarctica lon: 90.1 lat: 72.6 ang: 47.9
-53.187 -55.167
-52.993 -55.185
-52.886 -55.205
-52.829 -55.195
-52.740 -55.209
-52.637 -55.220
-52.489 -55.217
>Magnetic reversal anomaly edge in central Scotia Sea pre-Drake Passage location; rotation with respect to East Antarctica lon: 90.1 lat: 72.6 ang: 47.9
-52.210 -55.204
-51.763 -55.210
>Magnetic reversal anomaly edge in central Scotia Sea pre-Drake Passage location; rotation with respect to East Antarctica lon: 90.1 lat: 72.6 ang: 47.9
-53.226 -55.064
-53.044 -55.080
>Magnetic reversal anomaly edge in central Scotia Sea pre-Drake Passage location; rotation with respect to East Antarctica lon: 90.1 lat: 72.6 ang: 47.9
-52.852 -55.085
-52.700 -55.090
-52.559 -55.110
-52.487 -55.114
>Magnetic reversal anomaly edge in central Scotia Sea pre-Drake Passage location; rotation with respect to East Antarctica lon: 90.1 lat: 72.6 ang: 47.9
-52.355 -55.140
-52.127 -55.138
-52.008 -55.143
-51.898 -55.135
-51.764 -55.134
-51.663 -55.133
-51.585 -55.142
-51.492 -55.144
-51.399 -55.127
>Magnetic reversal anomaly edge in central Scotia Sea pre-Drake Passage location; rotation with respect to East Antarctica lon: 90.1 lat: 72.6 ang: 47.9
-53.196 -54.995
-53.008 -55.012
>Magnetic reversal anomaly edge in central Scotia Sea pre-Drake Passage location; rotation with respect to East Antarctica lon: 90.1 lat: 72.6 ang: 47.9

-52.830 -55.040
-52.635 -55.037
-52.544 -55.044
-52.456 -55.041
>Magnetic reversal anomaly edge in central Scotia Sea pre-Drake Passage location; rotation with respect to East Antarctica lon: 90.1 lat: 72.6 ang: 47.9
-52.386 -55.013
-52.195 -55.022
-52.085 -55.014
-51.926 -55.020
-51.794 -55.026
-51.695 -55.029
-51.621 -55.030
-51.445 -55.025
>Magnetic reversal anomaly edge in central Scotia Sea pre-Drake Passage location; rotation with respect to East Antarctica lon: 90.1 lat: 72.6 ang: 47.9
-52.710 -54.901
-52.525 -54.909
>Magnetic reversal anomaly edge in central Scotia Sea pre-Drake Passage location; rotation with respect to East Antarctica lon: 90.1 lat: 72.6 ang: 47.9
-52.447 -54.894
-52.276 -54.905
>Magnetic reversal anomaly edge in central Scotia Sea pre-Drake Passage location; rotation with respect to East Antarctica lon: 90.1 lat: 72.6 ang: 47.9
-52.098 -54.913
-51.927 -54.923
>Magnetic reversal anomaly edge in central Scotia Sea pre-Drake Passage location; rotation with respect to East Antarctica lon: 90.1 lat: 72.6 ang: 47.9
-52.728 -54.815
-52.534 -54.836
>Magnetic reversal anomaly edge in central Scotia Sea pre-Drake Passage location; rotation with respect to East Antarctica lon: 90.1 lat: 72.6 ang: 47.9
-52.441 -54.818
-52.263 -54.826
>Magnetic reversal anomaly edge in central Scotia Sea pre-Drake Passage location; rotation with respect to East Antarctica lon: 90.1 lat: 72.6 ang: 47.9
-52.086 -54.858
-51.901 -54.866
>Magnetic reversal anomaly edge in central Scotia Sea pre-Drake Passage location; rotation with respect to East Antarctica lon: 90.1 lat: 72.6 ang: 47.9
-52.147 -54.799
-51.962 -54.810
-51.933 -54.836
-51.766 -54.839
-51.676 -54.841
-51.643 -54.838
-51.635 -54.857

-51.496 -54.858
-51.361 -54.860
>Magnetic reversal anomaly edge in central Scotia Sea pre-Drake Passage location; rotation with respect to East Antarctica lon: 90.1 lat: 72.6 ang: 47.9
-52.134 -54.763
-52.029 -54.775
-51.949 -54.770
-51.920 -54.798
-51.751 -54.804
-51.707 -54.808
-51.669 -54.789
-51.576 -54.790
-51.385 -54.807
>Magnetic reversal anomaly edge in central Scotia Sea pre-Drake Passage location; rotation with respect to East Antarctica lon: 90.1 lat: 72.6 ang: 47.9
-52.085 -54.690
-51.973 -54.691
-51.957 -54.678
-51.840 -54.691
-51.805 -54.715
-51.652 -54.720
-51.493 -54.723
-51.419 -54.726
-51.309 -54.739
-51.236 -54.777
-51.173 -54.779
-51.108 -54.781
>Magnetic reversal anomaly edge in central Scotia Sea pre-Drake Passage location; rotation with respect to East Antarctica lon: 90.1 lat: 72.6 ang: 47.9
-52.071 -54.627
-51.996 -54.628
-51.933 -54.624
-51.884 -54.612
-51.738 -54.618
-51.612 -54.624
-51.590 -54.649
-51.465 -54.644
-51.336 -54.656
-51.316 -54.680
-51.176 -54.685
-51.141 -54.690
>Magnetic reversal anomaly edge in central Scotia Sea pre-Drake Passage location; rotation with respect to East Antarctica lon: 90.1 lat: 72.6 ang: 47.9
-51.894 -54.418
-51.702 -54.427
-51.560 -54.443
-51.429 -54.453

-51.283 -54.458
-51.048 -54.500
>Magnetic reversal anomaly edge in central Scotia Sea pre-Drake Passage location; rotation with respect to East Antarctica lon: 90.1 lat: 72.6 ang: 47.9
-51.348 -55.838
-51.159 -55.841
-50.932 -55.837
>Magnetic reversal anomaly edge in central Scotia Sea pre-Drake Passage location; rotation with respect to East Antarctica lon: 90.1 lat: 72.6 ang: 47.9
-51.242 -55.744
-51.094 -55.748
-50.915 -55.738
>Magnetic reversal anomaly edge in central Scotia Sea pre-Drake Passage location; rotation with respect to East Antarctica lon: 90.1 lat: 72.6 ang: 47.9
-51.311 -55.629
-51.185 -55.668
-51.066 -55.669
-50.985 -55.621
-50.829 -55.638
-50.726 -55.649
-50.599 -55.657
>Magnetic reversal anomaly edge in central Scotia Sea pre-Drake Passage location; rotation with respect to East Antarctica lon: 90.1 lat: 72.6 ang: 47.9
-51.316 -55.520
-51.212 -55.532
-51.109 -55.543
-50.996 -55.499
-50.833 -55.523
-50.600 -55.568
>Magnetic reversal anomaly edge in central Scotia Sea pre-Drake Passage location; rotation with respect to East Antarctica lon: 90.1 lat: 72.6 ang: 47.9
-51.711 -53.565
-51.624 -53.603
-51.566 -53.609
-51.436 -53.703
-51.370 -53.711
-51.246 -53.752
-51.136 -53.788
-51.005 -53.831
-50.914 -53.855
-50.838 -53.901
-50.780 -53.958
-50.675 -54.012
-50.600 -54.062
>Magnetic reversal anomaly edge in central Scotia Sea pre-Drake Passage location; rotation with respect to East Antarctica lon: 90.1 lat: 72.6 ang: 47.9
-51.618 -54.014

-51.558 -54.039
-51.501 -54.074
-51.403 -54.098
-51.345 -54.120
-51.243 -54.098
-51.120 -54.116
-51.011 -54.179
-50.910 -54.218
-50.833 -54.240
>Magnetic reversal anomaly edge in central Scotia Sea pre-Drake Passage location; rotation with respect to East Antarctica lon: 90.1 lat: 72.6 ang: 47.9
-51.641 -54.186
-51.547 -54.217
-51.472 -54.243
-51.390 -54.260
-51.318 -54.268
>Magnetic reversal anomaly edge in central Scotia Sea pre-Drake Passage location; rotation with respect to East Antarctica lon: 90.1 lat: 72.6 ang: 47.9
-51.685 -54.275
-51.433 -54.316
>Magnetic reversal anomaly edge in central Scotia Sea pre-Drake Passage location; rotation with respect to East Antarctica lon: 90.1 lat: 72.6 ang: 47.9
-50.969 -53.537
-50.869 -53.593
-50.773 -53.627
-50.709 -53.667
-50.597 -53.693
-50.476 -53.697
-50.476 -53.697
-50.466 -53.712
-50.343 -53.782
-50.262 -53.862
>Magnetic reversal anomaly edge in central Scotia Sea pre-Drake Passage location; rotation with respect to East Antarctica lon: 90.1 lat: 72.6 ang: 47.9
-50.901 -53.391
-50.772 -53.499
-50.607 -53.571
-50.246 -53.658
>Magnetic reversal anomaly edge in central Scotia Sea pre-Drake Passage location; rotation with respect to East Antarctica lon: 90.1 lat: 72.6 ang: 47.9
-50.291 -53.202
-50.033 -53.340
-49.666 -53.490
-49.437 -53.639
-49.208 -53.789
>Magnetic reversal anomaly edge in central Scotia Sea pre-Drake Passage location; rotation with respect to East Antarctica lon: 90.1 lat: 72.6 ang: 47.9

-51.009   -54.993
-50.816   -54.998
-50.666   -54.993
-50.595   -55.001
>Magnetic reversal anomaly edge in central Scotia Sea pre-Drake Passage location; rotation with respect to East Antarctica lon: 90.1 lat: 72.6 ang: 47.9
-51.282   -55.327
-51.141   -55.355
-50.977   -55.380
-50.852   -55.394
-50.736   -55.432
-50.608   -55.466
>Magnetic reversal anomaly edge in central Scotia Sea pre-Drake Passage location; rotation with respect to East Antarctica lon: 90.1 lat: 72.6 ang: 47.9
-51.246   -55.222
-51.087   -55.249
-50.654   -55.320
-50.492   -55.351
>Magnetic reversal anomaly edge in central Scotia Sea pre-Drake Passage location; rotation with respect to East Antarctica lon: 90.1 lat: 72.6 ang: 47.9
-50.472   -56.188
-50.678   -56.192
-50.842   -56.184
-50.990   -56.184
-51.063   -56.181
>Magnetic reversal anomaly edge in central Scotia Sea pre-Drake Passage location; rotation with respect to East Antarctica lon: 90.1 lat: 72.6 ang: 47.9
-49.871   -55.203
-49.705   -55.215
-49.628   -55.287
-49.449   -55.289
-49.296   -55.267
>Magnetic reversal anomaly edge in central Scotia Sea pre-Drake Passage location; rotation with respect to East Antarctica lon: 90.1 lat: 72.6 ang: 47.9
-49.749   -55.161
-49.610   -55.188
-49.457   -55.205
-49.294   -55.218
-49.201   -55.217
>Magnetic reversal anomaly edge in central Scotia Sea pre-Drake Passage location; rotation with respect to East Antarctica lon: 90.1 lat: 72.6 ang: 47.9
-49.676   -55.018
-49.467   -55.050
-49.392   -55.129
-49.240   -55.152
-49.082   -55.113
-48.944   -55.146

-48.794 -55.194
-48.661 -55.247
>Magnetic reversal anomaly edge in central Scotia Sea pre-Drake Passage location; rotation with respect to East Antarctica lon: 90.1 lat: 72.6 ang: 47.9
-49.486 -54.880
-49.314 -54.977
-49.230 -55.003
-49.087 -55.047
>Magnetic reversal anomaly edge in central Scotia Sea pre-Drake Passage location; rotation with respect to East Antarctica lon: 90.1 lat: 72.6 ang: 47.9
-49.224 -54.652
-49.125 -54.719
-48.816 -54.826
-48.688 -54.941
-48.569 -54.977
>Magnetic reversal anomaly edge in central Scotia Sea pre-Drake Passage location; rotation with respect to East Antarctica lon: 90.1 lat: 72.6 ang: 47.9
-51.440 -53.630
-51.535 -53.568
-51.601 -53.515
-51.686 -53.454
-51.793 -53.397
-51.952 -53.341
-52.063 -53.297
-52.258 -53.223
-52.381 -53.184
-52.503 -53.144
-52.624 -53.099
>Magnetic reversal anomaly edge in central Scotia Sea pre-Drake Passage location; rotation with respect to East Antarctica lon: 90.1 lat: 72.6 ang: 47.9
-51.095 -54.016
-51.141 -53.969
-51.242 -53.907
-51.462 -53.785
-51.542 -53.752
-51.632 -53.700
-51.748 -53.636
-52.010 -53.574
-52.171 -53.509
-52.290 -53.477
-52.406 -53.460
-52.501 -53.403
>Magnetic reversal anomaly edge in central Scotia Sea pre-Drake Passage location; rotation with respect to East Antarctica lon: 90.1 lat: 72.6 ang: 47.9
-51.813 -54.007
-51.889 -53.984
-52.053 -53.975

-52.126 -53.977
-52.216 -53.939
-52.280 -53.910
>Magnetic reversal anomaly edge in central Scotia Sea pre-Drake Passage location; rotation with respect to East Antarctica lon: 90.1 lat: 72.6 ang: 47.9
-51.985 -54.358
-52.208 -54.277
>Magnetic reversal anomaly edge in central Scotia Sea pre-Drake Passage location; rotation with respect to East Antarctica lon: 90.1 lat: 72.6 ang: 47.9
-50.889 -53.433
-51.081 -53.347
-51.180 -53.265
-51.334 -53.189
-51.441 -53.132
-51.550 -53.081
>Magnetic reversal anomaly edge in central Scotia Sea pre-Drake Passage location; rotation with respect to East Antarctica lon: 90.1 lat: 72.6 ang: 47.9
-49.370 -53.834
-49.521 -53.764
-49.693 -53.664
-49.850 -53.566
-49.980 -53.471
-50.190 -53.403
-50.378 -53.310
-50.519 -53.249
-50.780 -53.147
-50.917 -53.086
>Magnetic reversal anomaly edge in central Scotia Sea pre-Drake Passage location; rotation with respect to East Antarctica lon: 90.1 lat: 72.6 ang: 47.9
-52.545 -53.275
-52.375 -53.336
-52.287 -53.346
-52.203 -53.393
-52.103 -53.437
-51.991 -53.492
-51.872 -53.524
>Magnetic reversal anomaly edge in central Scotia Sea pre-Drake Passage location; rotation with respect to East Antarctica lon: 90.1 lat: 72.6 ang: 47.9
-53.823 -55.943
-53.960 -55.948
-53.972 -55.960
>Magnetic reversal anomaly edge in central Scotia Sea pre-Drake Passage location; rotation with respect to East Antarctica lon: 90.1 lat: 72.6 ang: 47.9
-54.708 -55.946
-54.564 -55.933
-54.400 -55.926
-54.301 -55.919

-54.257 -55.924
>Magnetic reversal anomaly edge in central Scotia Sea pre-Drake Passage location; rotation with respect to East Antarctica lon: 90.1 lat: 72.6 ang: 47.9
-49.870 -55.080
-50.127 -54.923
-50.153 -54.906
>Magnetic reversal anomaly edge in central Scotia Sea pre-Drake Passage location; rotation with respect to East Antarctica lon: 90.1 lat: 72.6 ang: 47.9
-50.122 -54.800
-50.261 -54.765
-50.315 -54.700
-50.340 -54.646
-50.438 -54.648
-50.438 -54.648
-50.457 -54.639
-50.511 -54.640
-50.567 -54.549
-50.673 -54.511
-50.819 -54.527
>Magnetic reversal anomaly edge in central Scotia Sea pre-Drake Passage location; rotation with respect to East Antarctica lon: 90.1 lat: 72.6 ang: 47.9
-49.576 -54.870
-49.721 -54.783
>Magnetic reversal anomaly edge in central Scotia Sea pre-Drake Passage location; rotation with respect to East Antarctica lon: 90.1 lat: 72.6 ang: 47.9
-49.990 -53.798
-49.912 -53.816
-49.831 -53.872
-49.753 -53.914
-49.701 -53.966
-49.607 -53.981
-49.518 -54.085
-49.466 -54.137
-49.356 -54.196
>Magnetic reversal anomaly edge in central Scotia Sea pre-Drake Passage location; rotation with respect to East Antarctica lon: 90.1 lat: 72.6 ang: 47.9
-50.921 -54.783
-50.645 -54.861
-50.615 -54.864
-50.599 -54.866
-50.578 -54.873
-50.564 -54.879
-50.557 -54.880
-50.549 -54.881
-50.534 -54.883
-50.535 -54.887
-50.528 -54.888

-50.522 -54.894
-50.515 -54.899
-50.509 -54.905
-50.412 -54.968
-50.277 -55.040
>Magnetic reversal anomaly edge in central Scotia Sea pre-Drake Passage location; rotation with respect to East Antarctica lon: 90.1 lat: 72.6 ang: 47.9
0.000 0.000
-52.253 -53.865
-52.157 -53.899
-52.089 -53.920
-51.866 -53.936
-51.806 -53.961
>Magnetic reversal anomaly edge in central Scotia Sea pre-Drake Passage location; rotation with respect to East Antarctica lon: 90.1 lat: 72.6 ang: 47.9
-51.635 -53.947
-51.525 -53.983
-51.447 -54.024
-51.440 -54.025
-51.432 -54.026
-51.425 -54.026
-51.417 -54.027
-51.403 -54.029
-51.396 -54.029
-51.388 -54.030
-51.381 -54.031
-51.374 -54.032
-51.366 -54.033
-51.359 -54.034
-51.352 -54.034
-51.350 -54.030
-51.336 -54.032
-51.328 -54.032
-51.313 -54.034
-51.306 -54.035
-51.299 -54.036
-51.291 -54.036
-51.233 -54.043
>Magnetic reversal anomaly edge in central Scotia Sea pre-Drake Passage location; rotation with respect to East Antarctica lon: 90.1 lat: 72.6 ang: 47.9
-51.734 -53.829
-51.741 -53.828
-51.747 -53.823
-51.745 -53.819
-51.753 -53.818
-51.751 -53.813
-51.759 -53.812

-51.765 -53.807
-51.772 -53.806
-51.770 -53.802
-51.829 -53.795
-52.200 -53.698
-52.279 -53.661
-52.360 -53.653
>Magnetic reversal anomaly edge in central Scotia Sea pre-Drake Passage location; rotation with respect to East Antarctica lon: 90.1 lat: 72.6 ang: 47.9
-52.334 -53.739
-52.268 -53.747
-52.246 -53.749
-52.232 -53.751
-52.224 -53.752
-52.211 -53.758
-52.198 -53.764
-52.191 -53.765
-52.185 -53.770
-52.179 -53.775
-51.893 -53.853
-51.881 -53.864
-51.874 -53.865
-51.867 -53.866
-51.861 -53.871
-51.853 -53.872
-51.855 -53.876
-51.847 -53.877
-51.786 -53.898
>Magnetic reversal anomaly edge in central Scotia Sea pre-Drake Passage location; rotation with respect to East Antarctica lon: 90.1 lat: 72.6 ang: 47.9
-52.417 -53.081
-52.313 -53.112
-52.284 -53.115
-52.276 -53.116
-52.269 -53.117
-52.262 -53.117
-52.247 -53.119
-52.240 -53.120
-52.233 -53.121
-52.225 -53.121
-51.995 -53.161
-51.910 -53.204
-51.889 -53.211
-51.877 -53.221
-51.864 -53.228
-51.856 -53.228
-51.851 -53.234

-51.782 -53.256
-51.724 -53.267
-51.717 -53.267
-51.710 -53.268
-51.702 -53.269
-51.629 -53.301
-51.609 -53.312
-51.598 -53.323
-51.592 -53.329
-51.572 -53.340
-51.598 -53.323
>Magnetic reversal anomaly edge in central Scotia Sea pre-Drake Passage location; rotation with respect to East Antarctica lon: 90.1 lat: 72.6 ang: 47.9
-51.518 -53.408
-51.450 -53.458
-51.438 -53.468
-51.426 -53.479
-51.420 -53.484
-51.408 -53.495
-51.395 -53.501
-51.388 -53.502
-51.382 -53.507
-51.367 -53.509
-51.368 -53.514
-51.361 -53.514
-51.355 -53.520
-51.295 -53.545
-51.211 -53.569
>
